# Marker aided introgression of ‘*Saltol’*, a major QTL for seedling stage salinity tolerance into an elite Basmati rice variety ‘Pusa Basmati 1509’

**DOI:** 10.1038/s41598-020-70664-0

**Published:** 2020-09-04

**Authors:** Ashutosh Kumar Yadav, Aruna Kumar, Nitasha Grover, Ranjith Kumar Ellur, S. Gopala Krishnan, Haritha Bollinedi, Prolay Kumar Bhowmick, K. K. Vinod, M. Nagarajan, S. L. Krishnamurthy, Ashok Kumar Singh

**Affiliations:** 1 Division of Genetics, ICAR-Indian Agricultural Research Institute, New Delhi, 110012 India; 2 Amity Institute of Biotechnology, Amity University, Noida, 201303 India; 3 Rice Breeding and Genetics Research Centre, ICAR-Indian Agricultural Research Institute, Aduthurai, Tamil Nadu 612101 India; 4 ICAR-Central Soil Salinity Research Institute, Karnal, 132001 India

**Keywords:** Plant biotechnology, Plant breeding

## Abstract

Marker assisted backcross breeding was used to transfer *Saltol*, a major QTL for seedling stage salinity tolerance from the donor FL478 to Pusa Basmati 1509 (PB 1509), a high yielding and early maturing Basmati rice variety. Foreground selection was carried out using three markers namely, AP3206f, RM3412b and RM10793, linked to *Saltol*. In addition, 105 genome-wide SSR markers polymorphic between FL478 and PB 1509 were used in background selection. Among the BC_3_F_4_ near isogenic lines (NILs) developed, recurrent parent genome recovery ranged from 96.67 to 98.57%. Multi-season evaluation identified some of the NILs showing significantly higher yield with grain and cooking quality comparable to PB 1509. All the NILs exhibited tolerance to salinity with significantly higher relative water content, membrane stability index and proline content as compared to PB 1509. The root and shoot concentration of Na^+^, K^+^ and Na^+^/K^+^ in NILs was at par with FL478 under stress conditions. The gene *OsHKT1*;5 located in the *Saltol* region showed higher expression levels under stress indicating its role in conferring salinity tolerance. Salt tolerant NILs of PB 1509 will be useful in stabilizing production in salt affected areas.

## Introduction

Rice is highly sensitive to salinity stress at seedling and reproductive stages. The symptoms of salt injury in rice are stunted growth, rolling of leaves, white tips, drying of older leaves and grain sterility. The most common injuries are attributed to the destabilization of the membrane, osmotic imbalance and disruption of photosynthetic mechanism^1,2^. Water uptake by rice plant is also hindered due to salt stress which causes leaf damage^3,4^. Soil salinity limits the rice plant’s growth and development, resulting in yield losses of more than 50%^5^. Though salinity affects all stages of the growth and development of the rice plant, its effect on young seedlings is highly detrimental as it directly influences plant establishment, thus affecting yield. With every dS/m increase of electrical conductivity (EC) beyond the threshold salt level of 3.0 dS/m, the rice yield is decreased by 12% which implies a yield reduction up to 50% at EC 7.2 dS/m^6^. Therefore, development of varieties with seedling stage salinity tolerance can sustain the production of the crop in salt affected areas by promoting the good initial establishment of plants, leading to healthy vegetative growth that can increase crop yield^7^. In India, rice is grown on 44 million ha with an annual production of 110 million tons of milled rice. Basmati is premium quality rice which is grown in ~ 2 million ha across seven states of India which has been earmarked as the Geographical Indication (GI) area for the cultivation^8^. Basmati rice is well-known worldwide for its exquisite quality traits, superfine grains, fluffy cooked rice with superior eating quality and pleasant aroma^9^. It is a valuable agricultural export commodity, which earned foreign exchange worth US$ 4.72 billion during 2018–2019^10^.

In India, about 7.0 million hectares (mha) is salt affected, of which a sizeable portion occurs in the Indo-Gangetic region comprising the states of Haryana, Punjab, and Uttar Pradesh where Basmati rice is majorly grown^11^. The inland salinity is a result of continuous use of underground brackish water for irrigation^12^. Therefore, developing genetic tolerance to salinity stress becomes imperative.

For seedling stage salinity tolerance, a major QTL, *Saltol*, explaining 43–70% of phenotypic variation was mapped on chromosome 1 in the population derived from a cross between IR29/Pokkali^13,14^. A seedling-stage salt tolerant recombinant inbred line from this cross, FL478 (IR66946-3R-178-1-1) has been widely used as a donor for enhancing salt tolerance in rice. Haplotype analysis of FL478 *Saltol* region revealed an introgression of < 1 Mb chromosomal segment (10.6–11.5 Mb region) from a tolerant parent Pokkali^15,16^. In another population generated from a cross Nona Bokra/Koshihikari, a QTL governing salinity stress was mapped to the same region^17^, which was further fine-mapped and cloned as *OsHKT1;5* which encodes a sodium transporter that maintains K^+^ homeostasis^18^.

Although, there are several reports of introgression of *Saltol* QTL into non-Basmati rice varieties like IR64, BR11, BRRI Dhan 28, and AS996^19–22^, only two high yielding Basmati rice varieties namely, Pusa Basmati 1^2^ and Pusa Basmati 1121^23^ have been improved for seedling stage salinity tolerance through MABB. Therefore, there is a need to improve other high yielding Basmati varieties for salinity tolerance to stabilize the Basmati rice production in salt affected soils. Pusa Basmati 1509 (PB 1509) developed by the ICAR-Indian Agricultural Research Institute (ICAR-IARI), New Delhi, India, is an early maturing Basmati rice variety with seed to seed maturity of 120 days, semi-dwarf plant stature with high yields and excellent grain and cooking quality traits^24^. However, this variety is sensitive to salinity at seedling as well as the reproductive stages. Here, we report introgression of *Saltol* QTL from donor parent (DP) FL478 into the recurrent parent (RP) PB 1509 using Marker assisted backcross breeding (MABB) approach.

## Results

### Marker assisted introgression of *Saltol* into PB 1509

**Figure 1:**
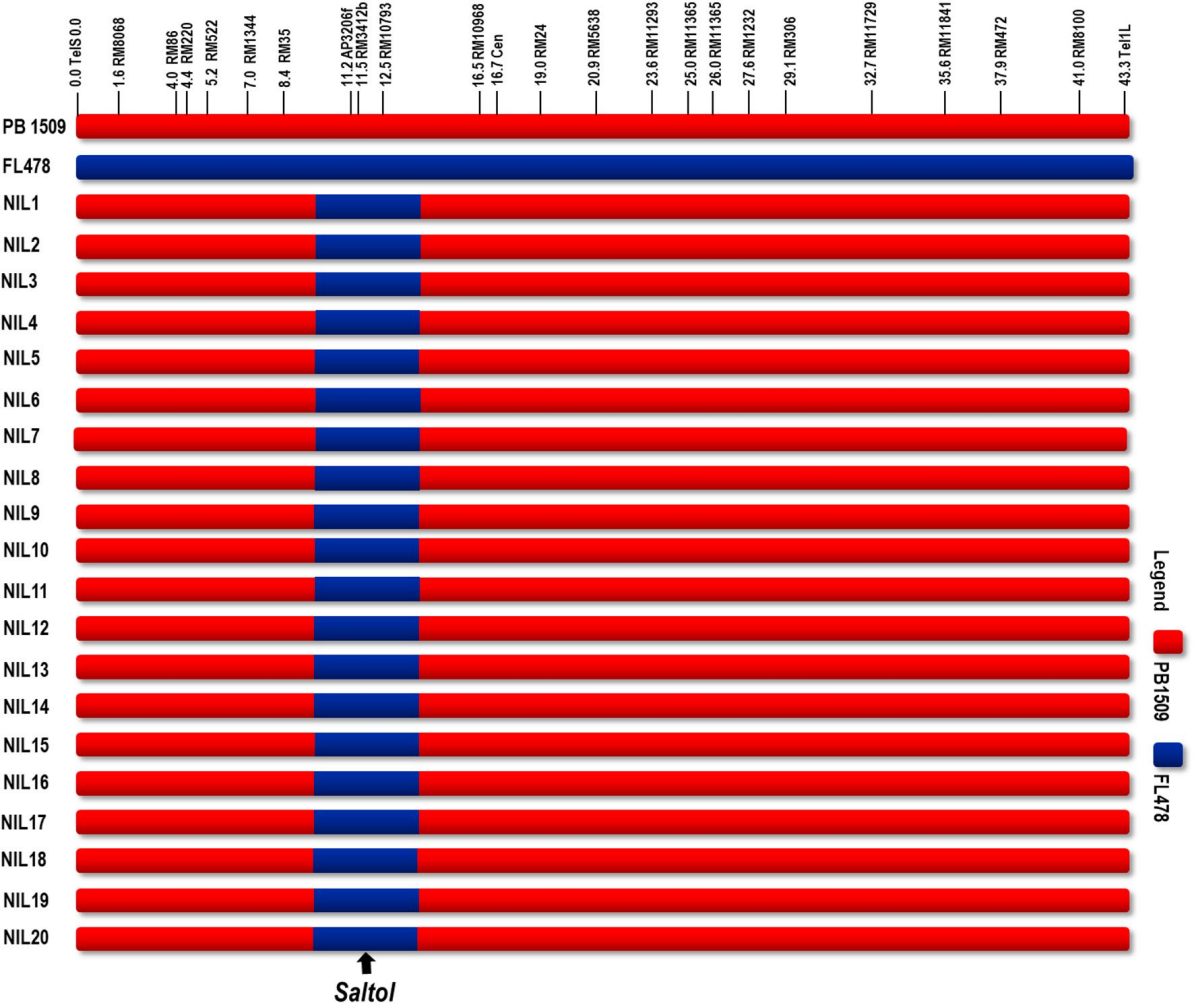
Graphical representation of PB 1509-NILs carrying ‘*Saltol’* depicting the extent of recovery of carrier chromosome (Chromosome 1).

Under hydroponic screening with 120 mM NaCl (EC of 13.9 dS/m), the seedlings of PB 1509 were highly sensitive with a score of 9, while FL478 was highly tolerant with a score of 1 (Supplementary Fig. S1). MABB was used to transfer *Saltol* from FL478 into PB 1509. The F_1_s generated from the cross PB 1509/FL478 were tested for hybridity with *Saltol* linked markers viz*.* AP3206f, RM3412 and RM10793. A single F_1_ plant heterozygous for all three foreground markers was backcrossed with the RP and 388 BC_1_F_1_ seeds were generated, of which 162 plants were heterozygous for three foreground markers. The background selection among 162 *Saltol* positive plants with 105 genome-wide SSR markers revealed the recurrent parent genome (RPG) recovery ranging from 77.62 to 81.9%. Further, phenotypic selection for agro-morphological traits was carried out to identify the BC_1_F_1_ with maximum similarity to the RP. Based on background and phenotypic selection, a BC_1_F_1_ plant, Pusa 1960-3 with 81.90% RPG was selected and backcrossed with the PB 1509 and 128 BC_2_F_1_ seeds were produced. A total of 48 BC_2_F_1_ plants were heterozygous for all three foreground markers with RPG recovery ranging from 89.52 to 93.33%. A BC_2_F_1_ plant with maximum RPG and recurrent parent phenome (RPP) recovery was further backcrossed and 66 BC_3_F_1_ seeds were produced. Of these, one desirable BC_3_F_1_ plant heterozygous for *Saltol* with maximum RPG recovery (96.19%) was selfed to produce BC_3_F_2_ population. Foreground selection was carried out on 460 BC_3_F_2_ plants and a total of 108 plants were found to be homozygous for three markers linked to *Saltol* (Table 1). These progenies were evaluated for grain and cooking quality parameters during BC_3_F_3_ generation and 58 families were selected. Finally, based on seedling stage salinity tolerance, 20 highly tolerant near isogenic lines (NILs) were selected. The RPG recovery in the 20 selected NILs ranged from 96.67 to 98.57% with some residual donor segments in chromosomes 3, 5, 7, 8 and 12. While, there was complete recovery in carrier chromosome (Chromosome 1) together with the target QTL *Saltol* (Fig. 1).

**Table 1:** Number of plants generated and recurrent parent genome recovered in backcross generations during the marker aided introgression of *Saltol* QTL in PB 1509.

Generation	No. of plants			Genome recovery (%)
	Generated	*Saltol* positives^b^	Selected	
F_1_	25	22	1	^a^
BC_1_F_1_	388	162	1	77.62–81.90
BC_2_F_1_	128	48	1	89.52–93.33
BC_3_F_1_	66	28	1	94.76–96.19
BC_3_F_2_	460	108	58	^a^
BC_3_F_3_	58	58	20	^a^
BC_3_F_4_	20	20	20	96.67–98.57

^a^Not estimated.

^b^*Saltol* positives indicate the number of plants that were found to carry the target foreground marker alleles for *Saltol* QTL.

### Screening for seedling stage salt tolerance

Fifty eight BC_3_F_3_ families were screened for seedling stage salinity tolerance under hydroponics with salt concentration of 120 mM (EC of 13.9 dS/m) along with the parents (PB 1509 and FL478) and a susceptible check IR29. All the 58 lines had uniform growth under unstressed condition. However, under salt stress conditions, 28 families were found highly tolerant with standard evaluation system (SES) score of 1 as similar to the DP, FL478, and 30 families showed moderate tolerance with a score of 5–6. Finally, a set of 20 highly tolerant families (NIL 1–NIL 20) having superior grain and cooking quality traits and maximum RPG recovery were selected and advanced to BC_3_F_4_ generation (Supplementary Fig. S2).

**Figure 2:**
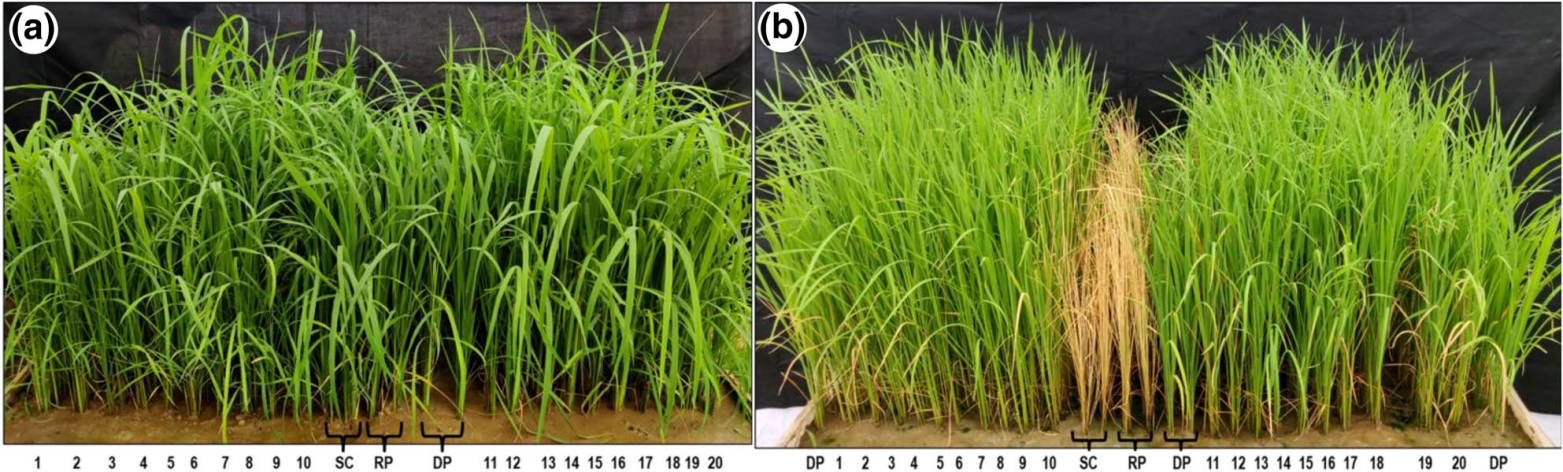
Screening of *Saltol*-introgressed PB 1509-NILs (1-20) along with the recurrent parent (RP) PB 1509, susceptible check (SC) IR29 and the donor parent (DP) FL 478 for seedling stage salinity tolerance under field condition (micro-plot) at EC of 13.9 dS/m, (**a**) unstressed, (**b**) salt-stressed condition.

These 20 NILs were further screened for seedling stage salinity tolerance under field conditions in the micro-plots at EC of 13.9 dS/m. Thirteen NILs, namely, NIL1, NIL2, NIL3, NIL4, NIL5, NIL6, NIL7, NIL8, NIL13, NIL14, NIL15, NIL16 and NIL18 were found highly tolerant with a SES score of one and seven NILs, namely, NIL9, NIL10, NIL11, NIL12, NIL17, NIL19 and NIL20 were found to be tolerant with a score of 3 (Fig. 2).

### Estimation of leaf RWC, MSI and proline content

**Figure 3:**
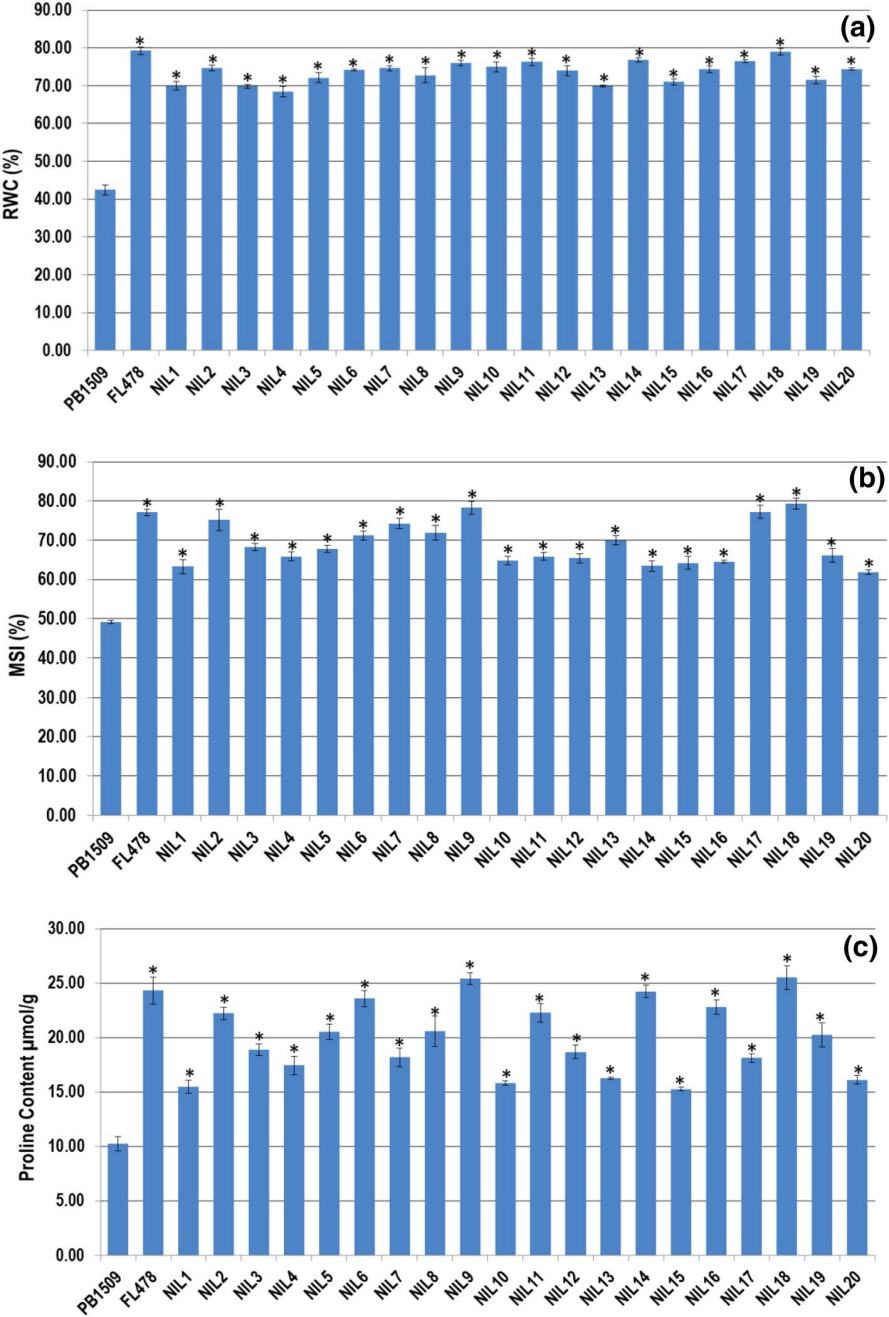
Effect of salt stress on parental lines and PB 1509-NILs for, (**a**) RWC: relative water content, (**b**) MSI: membrane stability index, and (**c**) Proline content. *Student’s* t-test was performed for statistical significance. An asterisk (*) above the bars refers significant difference from the RP-PB 1509 at *P* ≤ 0.05. The values represented are the mean of three biological replicates and standard error is shown as error bars.

The data on relative water content (RWC), membrane stability index (MSI) and proline content in parents and NILs are presented in Supplementary Table S4 and Fig. 3. Under unstressed conditions, average RWC of RP, DP and NILs was found to be 84.47, 91.80 and 81.92%, respectively, while RWC in NILs ranged from 78.24 to 86.73%. However, under salt stress conditions, the RWC of NILs ranged from 68.41% (NIL4) to 78.98% (NIL18), which was significantly superior to the RP (42.46%). Only four NILs (NIL11, NIL14, NIL17 and NIL 18) showed RWC in the range of 76.26–78.98%, which was statistically (Critical difference (CD) of 2.96 at 5%) at par with the DP (79.21%). Under unstressed conditions, MSI was more than 81% among parental lines and NILs. However, under salt stress conditions, the average MSI in NILs was found to be 68.92% with a range from 61.84 (NIL20) to 79.25% (NIL18), which was significantly higher than the RP (49.13%). However, only five NILs (NIL2, NIL7, NIL9, NIL17, and NIL18) showed MSI in the range of 74.27–79.25% which were statistically at par with the DP (77.08%).

The proline accumulation under unstressed conditions ranged from 6.44 to 8.76 µmol/g among the NILs with 8.41 µmol/g in DP and 7.54 µmol/g in RP. However, under stressed conditions, seven NILs (NIL2, NIL6, NIL9, NIL11, NIL14, NIL16 and NIL18) showed proline content at par with FL478, while rest of the NILs had proline content lower than the FL478 but significantly higher than PB 1509.

### Na^+^ and K^+^ content in shoot and root

The salt stress response in rice seedlings is majorly influenced by Na^+^ and K^+^ concentrations in root and shoot. Significant variation was observed among the parents and NILs for the cation content and their ratio in shoot and root under salt stressed conditions. Whereas under unstressed conditions, in both root and shoot, the Na^+^ and K^+^ concentrations and their ratio showed no apparent difference among the parents and NILs (Supplementary Table S5). Under salt stressed conditions, the Na^+^ concentration of RP (2.19 mmol/g of shoot dry weight and 3.33 mmol/g of root dry weight) was significantly higher than the DP (0.50 mmol/g of shoot dry weight and 0.73 mmol/g of root dry weight). The average shoot Na^+^ concentration among the NILs was 0.66 mmol/g of dry weight which ranged from 0.44 (NIL18) to 0.77 (NIL17) mmol/g of dry weight. This was significantly lower than the RP but comparable to DP. However, the average root Na^+^ concentration in NILs was 0.89 mmol/g of dry weight which ranged from 0.62 (NIL13) to 0.98 (NIL11) mmol/g of dry weight and was significantly lower than RP (3.33 mmol/g of dry weight) while was at par with DP.

Under salt stress, the average shoot K^+^ concentration in PB 1509, FL478 and NILs was 0.47, 1.27 and 1.05 mmol/g of shoot dry weight, respectively, while all the NILs exhibited significantly higher shoot K^+^ concentration ranging from 0.92 (NIL10) to 1.25 (NIL18) mmol/g of shoot dry weight than RP but statistically similar to that of the DP. The root K^+^ concentration in RP and DP ranged from 0.45 to 1.13 mmol/g of root dry weight, respectively. However, all the NILs exhibited significantly higher K^+^ concentration that ranged from 0.90 (NIL14) to 1.32 (NIL7) mmol/g of shoot dry weight as compared to RP, which remained similar to that of the DP.

**Figure 4:**
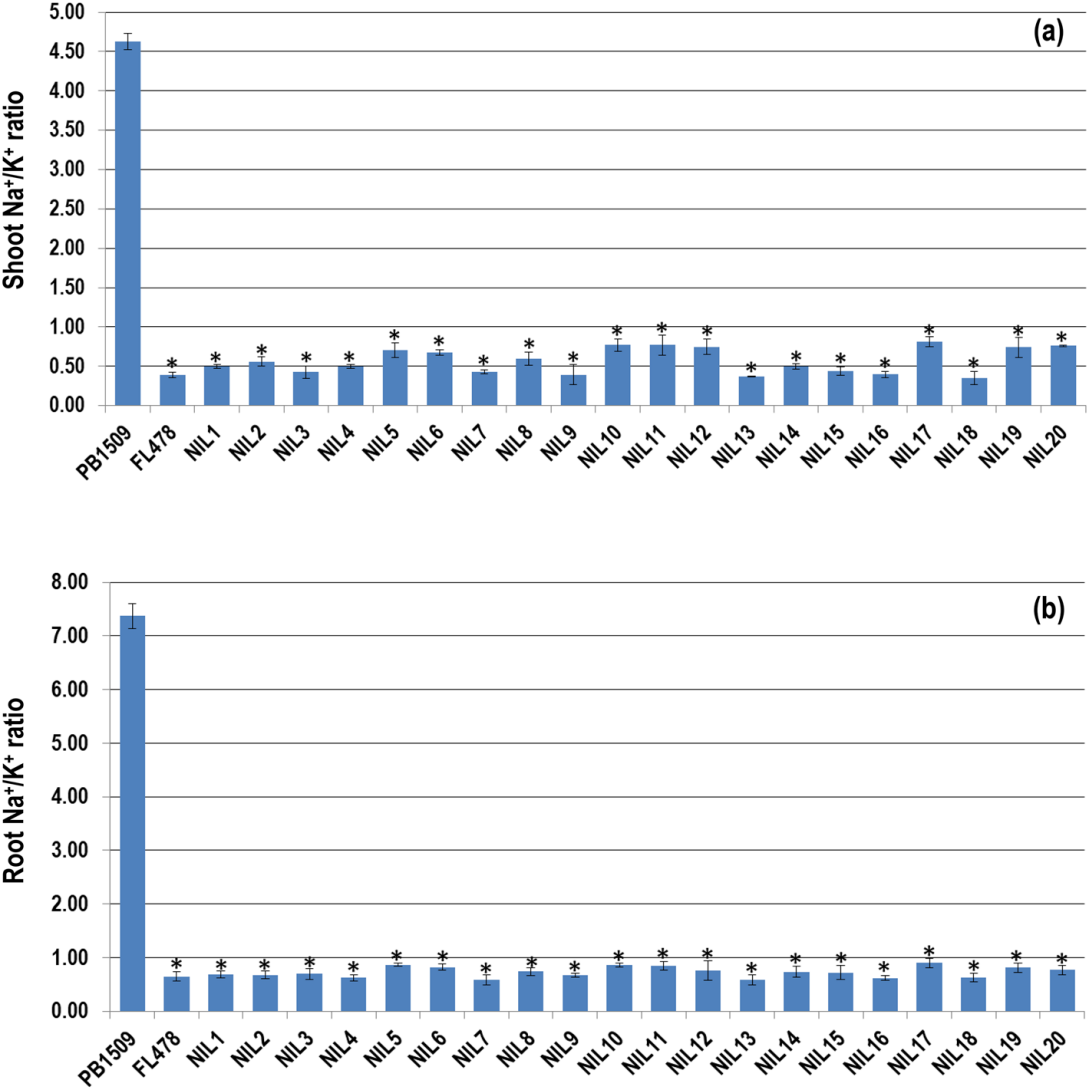
(**a**) Shoot and (**b**) root Na^+^/K^+^ ratio of PB 1509-NILs compared with recurrent and donor parents under salt stress condition. *Student’s* t-test was performed to test the statistical significance. An asterisk (*) above the bars refers to the significant differences from PB 1509 at *P* ≤ 0.05. The values represented are the mean of three biological replicates. Standard error is shown as error bars.

Under the unstressed condition, no significant difference was observed for Na^+^/K^+^ concentration in shoot and root. However, under salt stressed conditions the shoot Na^+^/K^+^ ratio of all the NILs ranged from 0.35 (NIL18) to 0.81 (NIL17) with an average of 0.75 which was significantly lower than that of the RP (4.62) and similar as that of the DP (0.39) (Fig. 4a). The root Na^+^/K^+^ ratio of NILs ranged from 0.58 (NIL13) to 0.90 (NIL17) with an average of 1.03 which was significantly lower than RP (7.37) but comparable to FL478 (0.65) (Fig. 4b).

### Correlation among physiological traits

The correlation between physiological traits and cation concentrations in shoot and root under salt stressed condition is presented in Supplementary Table 6. A significant negative correlation was found between salt tolerance score (STS) with MSI, RWC%, proline content, shoot K^+^ concentration and root K^+^ concentration, while there were significant positive correlations with root and shoot Na^+^ and Na^+^/K^+^ under salt stress. There was a significant positive correlation between MSI, RWC, and proline content. However, MSI, RWC and proline content were negatively correlated with the shoot and root Na^+^ and Na^+^/K ratio, while positively correlated with the shoot and root K^+^ concentration.

### Agronomic performance and grain quality assessment

The mean performance of each of the 20 NILs for agronomic traits is presented in Table 2. All the PB 1509-NILs tested were similar to RP with exceptions for few traits. A field view of PB 1509 and one of its closely resembling NILs, Pusa 1960-3-25-7-423 (NIL 18) is presented in Supplementary Fig. S3. Among the NILs, however, NIL12 and NIL17 were significantly late maturing; NIL9, NIL16 and NIL20 were significantly taller than PB 1509, while two other NILs, namely NIL9 and NIL18 possessed significantly higher spikelet fertility.

**Table 2:** Agronomic performance, salt tolerance score and RPG recovery (%) of PB 1509-NILs in comparison with RP (PB 1509) and DP (FL478).

NILs	Genotype	DFF	PH	NT	PL	SF	TW	STS	RPG
NIL1	Pusa 1960-3-25-7-3	86.0 ± 1.0	101.1 ± 3.18	12.5 ± 2.12	25.1 ± 0.78	83.7 ± 2.04	26.4 ± 0.47	1	97.62
NIL2	Pusa 1960-3-25-7-8	81.5 ± 2.0	103.4 ± 2.83	15.6 ± 0.57	30.0 ± 0.17	84.0 ± 6.10	28.7 ± 0.87	1	97.62
NIL3	Pusa 1960-3-25-7-25	81.0 ± 1.0	102.7 ± 2.76	15.2 ± 2.55	31.1 ± 0.31*	85.6 ± 10.8	27.5 ± 0.74	1	98.57
NIL4	Pusa 1960-3-25-7-42	83.5 ± 2.0	100.0 ± 4.81	15.0 ± 2.83	29.7 ± 0.37	84.9 ± 3.64	25.7 ± 0.79	1	98.57
NIL5	Pusa 1960-3-25-7-55	82.5 ± 2.0	99.5 ± 4.03	16.2 ± 0.28	29.6 ± 1.46	85.7 ± 0.60	28.5 ± 1.11	1	97.61
NIL6	Pusa 1960-3-25-7-122	84.5 ± 1.0	101.6 ± 3.34	13.7 ± 0.99	27.0 ± 0.99	86.5 ± 1.85	27.9 ± 2.37	1	97.14
NIL7	Pusa 1960-3-25-7-128	83.5 ± 1.0	95.7 ± 4.60	11.9 ± 2.83	30.1 ± 1.48	82.5 ± 1.92	27.8 ± 2.20	1	97.61
NIL8	Pusa 1960-3-25-7-162	85.0 ± 1.0	99.6 ± 3.96	11.1 ± 1.41	27.6 ± 0.23	80.5 ± 1.58	29.4 ± 1.11	1	97.14
NIL9	Pusa 1960-3-25-7-205	84.0 ± 1.0	104.9 ± 1.84*	16.0 ± 2.83	29.1 ± 0.17	87.3 ± 7.35*	27.6 ± 3.22	3	98.57
NIL10	Pusa 1960-3-25-7-246	84.0 ± 1.0	91.4 ± 1.56	12.9 ± 1.27	29.5 ± 0.38	83.3 ± 0.70	27.7 ± 2.76	3	98.10
NIL11	Pusa 1960-3-25-7-264	84.5 ± 2.0	102.1 ± 3.54	10.4 ± 0.57	27.5 ± 0.40	84.6 ± 7.06	27.3 ± 0.11	3	98.57
NIL12	Pusa 1960-3-25-7-290	87.0 ± 1.0*	91.9 ± 1.84	14.3 ± 2.40	26.9 ± 0.06	81.2 ± 2.01	29.7 ± 0.31	3	96.67
NIL13	Pusa 1960-3-25-7-296	83.5 ± 1.0	101.4 ± 1.63	12.4 ± 0.57	28.6 ± 0.72	79.2 ± 3.84	29.1 ± .077	1	97.14
NIL14	Pusa 1960-3-25-7-366	84.5 ± 1.0	95.5 ± 1.84	12.1 ± 1.27	28.2 ± 0.52	66.4 ± 1.32	26.1 ± 1.10	1	98.10
NIL15	Pusa 1960-3-25-7-380	80.5 ± 1.0	100.6 ± 0.85	13.2 ± 1.70	29.1 ± 0.24	82.3 ± 0.96	28.6 ± 1.57	1	98.10
NIL16	Pusa 1960-3-25-7-404	82.5 ± 2.0	105.4 ± 5.59*	15.3 ± 0.42	28.7 ± 0.31	82.6 ± 3.84	29.3 ± 0.78	1	97.62
NIL17	Pusa 1960-3-25-7-416	92.5 ± 2.0*	99.4 ± 2.83	13.3 ± 1.70	28.9 ± 0.20	80.3 ± 1.68	25.3 ± 1.20	3	97.62
NIL18	Pusa 1960-3-25-7-423	85.0 ± 1.0	95.0 ± 2.05	16.8 ± 1.42	29.7 ± 0.79	87.5 ± 1.44*	26.2 ± 1.58	1	98.10
NIL19	Pusa 1960-3-25-7-450	82.5 ± 2.0	97.7 ± 4.38	14.3 ± 1.84	26.1 ± 1.34	76.9 ± 3.04	28.6 ± 1.87	3	98.57
NIL20	Pusa 1960-3-25-7-458	85.5 ± 2.0	103.3 ± 3.54*	15.5 ± 1.27	27.9 ± 1.58	84.8 ± 1.67	28.3 ± 2.97	3	98.57
PB 1509	–	83.5 ± 1.0	96.6 ± 2.90	15.5 ± 0.71	29.5 ± 0.44	80.7 ± 1.16	27.6 ± 0.80	9	–
FL478	–	85.0 ± 1.0	97.5 ± 1.56	13.4 ± 0.57	26.3 ± 0.98	85.2 ± 1.48	24.9 ± 0.49	1	–
**CD(0.05)**		**2.77**	**6.50**	**3.51**	**1.60**	**6.50**	**3.35**		

DFF, days to 50% flowering; PH, plant height in cm; NT, number of effective tillers; PL, panicle length in cm; SF, spikelet fertility in %; TW, thousand grain weight in g; STS, salt tolerance score under field condition (micro-plot); RPG, recurrent parent genome recovery in %; CD: critical difference.

*Significance at 5%.

The mean grain and cooking quality characteristics of PB 1509-NILs are presented in Table 3. Hulling recovery (HUL), milling recovery (MIL) and head rice recovery (HRR) for all the NILs were similar to RP. All the grain quality parameters were comparable to the RP with the NILs possessing extra-long slender grain type. The cooking quality of the seven NILs namely NIL9, NIL12, NIL20, NIL13, NIL6, NIL10 and NIL19 were significantly superior with higher KLAC as compared to RP (Supplementary Fig. S4). All the NILs possessed strong aroma with an alkali spreading value (ASV) of 6 which was comparable to RP.

**Table 3:** Grain and cooking quality traits of PB 1509–*Saltol* NILs in comparison to PB 1509.

NILs	HUL	MIL	HRR	KLBC	KBBC	KLAC	KBAC	ER	ASV	AROMA
NIL1	76.8 ± 2.4	62.9 ± 1.7	48.8 ± 3.5	8.67 ± 0.28	1.58 ± 0.03	18.67 ± 0.42	2.05 ± 0.07	2.16 ± 0.12	6.00	2.0
NIL2	78.1 ± 2.1	63.6 ± 1.1	52.1 ± 1.9	8.50 ± 0.24	1.67 ± 0.14	18.20 ± 0.32	2.00 ± 0.00	2.15 ± 0.10	6.00	2.0
NIL3	80.9 ± 3.9	64.5 ± 3.4	50.9 ± 2.6	8.51 ± 0.24	1.71 ± 0.07	18.88 ± 0.64	2.28 ± 0.07*	2.22 ± 0.14	6.00	2.0
NIL4	81.7 ± 1.2	66.9 ± 2.2	51.2 ± 2.8	8.50 ± 0.71	1.50 ± 0.04	18.71 ± 0.40	2.05 ± 0.07	2.21 ± 0.14	6.00	2.0
NIL5	79.3 ± 1.8	66.1 ± 1.2	49.8 ± 5.1	8.84 ± 0.24	1.67 ± 0.14	18.98 ± 0.35	1.95 ± 0.07	2.15 ± 0.02	6.00	2.0
NIL6	77.7 ± 1.3	61.3 ± 0.9	51.4 ± 1.2	8.84 ± 0.24	1.64 ± 0.03	20.03 ± 0.42*	1.95 ± 0.07	2.27 ± 0.01*	6.00	2.0
NIL7	76.1 ± 0.6	63.9 ± 1.9	52.9 ± 2.2	8.67 ± 0.14	1.67 ± 0.03	18.98 ± 0.49	1.72 ± 0.07	2.19 ± 0.02	6.00	2.0
NIL8	78.7 ± 1.8	68.1 ± 3.3	50.4 ± 4.6	8.38 ± 0.07	1.67 ± 0.14	18.77 ± 0.14	2.00 ± 0.00	2.24 ± 0.00	6.00	2.0
NIL9	79.2 ± 0.9	67.2 ± 2.4	54.5 ± 2.6	8.88 ± 0.16	1.62 ± 0.03	21.47 ± 0.28*	2.28 ± 0.07*	2.42 ± 0.01*	6.00	2.0
NIL10	80.6 ± 2.5	66.0 ± 0.9	51.3 ± 4.4	8.84 ± 0.24	1.66 ± 0.01	19.67 ± 0.42*	1.95 ± 0.07	2.23 ± 0.01	6.00	2.0
NIL11	81.2 ± 1.7	67.2 ± 2.3	50.7 ± 1.6	8.43 ± 0.14	1.62 ± 0.06	19.00 ± 0.95	2.23 ± 0.14	2.25 ± 0.07	6.00	2.0
NIL12	81.1 ± 1.7	67.9 ± 3.5	51.3 ± 1.6	8.62 ± 0.07	1.62 ± 0.07	21.02 ± 0.28*	1.95 ± 0.21	2.49 ± 0.01*	6.00	2.0
NIL13	79.8 ± 0.8	65.3 ± 2.2	53.2 ± 2.3	8.84 ± 0.24	1.54 ± 0.02	20.08 ± 0.35*	2.10 ± 0.14	2.27 ± 0.10*	6.00	2.0
NIL14	78.5 ± 3.3	66.0 ± 0.6	51.5 ± 1.9	8.93 ± 0.09	1.66 ± 0.01	18.32 ± 0.49	2.05 ± 0.07	2.05 ± 0.03	6.00	2.0
NIL15	79.1 ± 2.0	65.1 ± 1.0	52.4 ± 1.4	8.23 ± 0.33	1.59 ± 0.06	18.92 ± 0.35	1.85 ± 0.21	2.30 ± 0.05*	6.00	2.0
NIL16	79.9 ± 2.0	69.1 ± 0.7	49.4 ± 3.6	8.77 ± 0.14	1.64 ± 0.02	18.73 ± 0.39	2.05 ± 0.07	2.14 ± 0.08	6.00	2.0
NIL17	82.0 ± 0.6	69.9 ± 0.5	48.5 ± 1.1	8.43 ± 0.14	1.65 ± 0.04	19.10 ± 0.33	2.05 ± 0.07	2.27 ± 0.08*	6.00	2.0
NIL18	78.4 ± 1.5	65.3 ± 2.3	53.1 ± 3.5	8.77 ± 0.14	1.67 ± 0.07	19.03 ± 0.42	2.28 ± 0.07*	2.17 ± 0.08	6.00	2.0
NIL19	80.8 ± 2.3	68.4 ± 1.2	50.1 ± 2.2	8.43 ± 0.14	1.59 ± 0.04	19.67 ± 0.42*	2.05 ± 0.07	2.33 ± 0.01*	6.00	2.0
NIL20	78.8 ± 3.8	67.2 ± 3.2	51.9 ± 1.1	8.96 ± 0.05	1.67 ± 0.14	20.98 ± 0.47*	1.85 ± 0.21	2.34 ± 0.07*	6.00	2.0
PB 1509	78.8 ± 2.5	68.0 ± 3.4	51.3 ± 2.8	8.84 ± 0.24	1.65 ± 0.03	18.43 ± 0.14	2.05 ± 0.07	2.10 ± 0.04	6.00	2.0
FL478	81.2 ± 3.9	67.5 ± 1.5	46.2 ± 1.5	6.60 ± 0.09	2.12 ± 0.16	10.77 ± 0.14	2.62 ± 0.07*	1.63 ± 0.04	5.00	0.0
**CD (0.05)**	**4.40**	**4.1**	**5.83**	**0.48**	**0.16**	**0.89**	**0.22**	**0.14**	**–**	**–**

HUL, hulling recovery in percentage; MIL, milling recovery in percentage; HRR, head rice recovery in percentage; KLBC: kernel length before cooking in mm; KBBC, kernel breadth before cooking in mm; KLAC, kernel length after cooking in mm; KBAC, kernel breath after cooking in mm; ER, elongation ratio; ASV, alkali spreading value; AROMA, aroma score from panel test; CD, critical difference.

*Significance at 5%.

### Multi-season evaluation and stability analysis for yield performance

All the PB 1509-NILs along with the parents were evaluated for three consecutive *Kharif* seasons between 2017 and 2019 and the yield data is presented in Table 4. During 2017, six NILs (NIL2, NIL3, NIL5, NIL9, NIL18 and NIL20) yielded significantly higher than PB 1509, while four NILs (NIL2, NIL3, NIL16 and NIL18) showed significantly superior yield performance during 2018. During 2019 season, five NILs (NIL3, NIL5, NIL7, NIL9, and NIL18) yielded significantly better than PB 1509. In all the three seasons, NIL3 and NIL18 performed consistently superior than the RP, while the other NILs performed at par.

**Table 4:** Performance of PB 1509-NILs for yield across three cropping seasons.

NILs	Genotype	Grain yield (kg/ha)			
		Kh 2017	Kh 2018	Kh 2019	Mean
NIL1	Pusa 1960-3-25-7-3	5,514.70 ± 203.81	5,472.22 ± 196.42	5,575.00 ± 141.42	5,520.64 ± 148.89
NIL2	Pusa 1960-3-25-7-8	5,867.65 ± 478.34*	5,777.78 ± 157.13*	5,775.00 ± 247.49	5,806.81 ± 255.29
NIL3	Pusa 1960-3-25-7-25	5,955.90 ± 395.15*	5,833.33 ± 117.85*	6,012.50 ± 265.17*	5,933.90 ± 234.05*
NIL4	Pusa 1960-3-25-7-42	5,426.50 ± 220.45	5,305.56 ± 127.85	5,650.00 ± 212.13	5,460.68 ± 214.31
NIL5	Pusa 1960-3-25-7-55	6,000.00 ± 207.97*	5,625.00 ± 255.34	5,987.50 ± 159.10*	5,870.83 ± 251.09*
NIL6	Pusa 1960-3-25-7-122	5,029.40 ± 224.61	5,152.78 ± 216.06	5,462.50 ± 194.45	5,214.90 ± 258.49
NIL7	Pusa 1960-3-25-7-128	5,602.95 ± 395.15	5,291.67 ± 373.20	6,162.50 ± 159.10*	5,685.70 ± 468.96*
NIL8	Pusa 1960-3-25-7-162	5,455.90 ± 220.47	5,625.00 ± 294.63	5,425.00 ± 247.49	5,501.97 ± 220.47
NIL9	Pusa 1960-3-25-7-205	6,102.95 ± 519.93*	5,597.22 ± 294.63	6,187.50 ± 265.17*	5,962.55 ± 408.66*
NIL10	Pusa 1960-3-25-7-246	5,338.25 ± 270.36	5,041.67 ± 255.34	5,450.00 ± 176.78	5,276.63 ± 263.02
NIL11	Pusa 1960-3-25-7-264	5,451.80 ± 241.25	5,111.11 ± 157.13	5,525.00 ± 282.84	5,349.29 ± 264.00
NIL12	Pusa 1960-3-25-7-290	5,529.45 ± 166.38	5,222.22 ± 187.13	5,675.00 ± 318.20	5,475.54 ± 271.05
NIL13	Pusa 1960-3-25-7-296	5,308.80 ± 203.81	5,291.67 ± 333.91	5,412.50 ± 335.88	5,337.66 ± 237.88
NIL14	Pusa 1960-3-25-7-366	5,441.15 ± 166.38	5,138.89 ± 157.13	5,537.50 ± 300.52	5,372.52 ± 251.28
NIL15	Pusa 1960-3-25-7-380	5,411.75 ± 499.13	5,250.00 ± 274.99	5,587.50 ± 123.74	5,416.42 ± 301.34
NIL16	Pusa 1960-3-25-7-404	5,676.50 ± 374.35	5,680.56 ± 98.21*	5,512.50 ± 194.45	5,623.18 ± 211.83*
NIL17	Pusa 1960-3-25-7-416	5,161.75 ± 104.0	5,013.89 ± 294.63	5,625.00 ± 282.84	5,266.88 ± 341.83
NIL18	Pusa 1960-3-25-7-423	6,014.70 ± 145.58*	5,833.33 ± 235.70*	6,237.50 ± 106.07*	6,028.51 ± 242.27*
NIL19	Pusa 1960-3-25-7-450	5,720.55 ± 145.58	5,583.33 ± 353.55	5,750.00 ± 229.81	5,684.64 ± 194.47*
NIL20	Pusa 1960-3-25-7-458	5,844.15 ± 137.27*	5,500.00 ± 196.42	5,687.50 ± 229.81	5,677.20 ± 213.99*
PB 1509	–	5,226.45 ± 195.49	5,166.67 ± 137.13	5,375.00 ± 116.07	5,256.05 ± 155.04
FL478	–	4,567.65 ± 137.26	4,680.56 ± 255.34	4,637.50 ± 229.81	4,628.57 ± 149.89
**CD (0.05)**		**587.34**	**512.10**	**452.37**	**287.27**

Kh, *Kharif* season.

*Significance at 5%.

**Figure 5:**
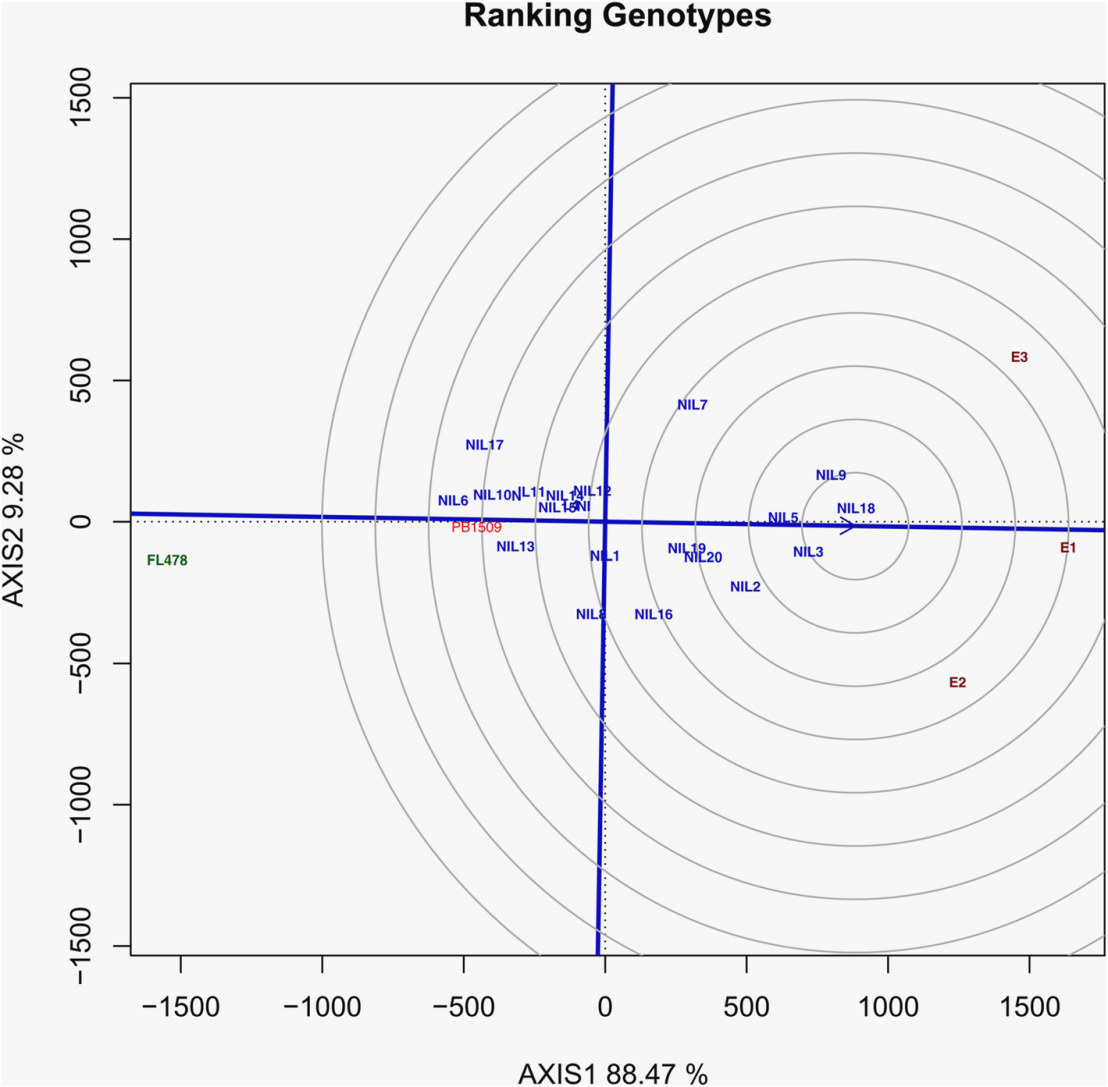
Ranking of PB 1509-NILs carrying *Saltol* QTL and PB 1509 and FL478 relative to an “ideal genotype”. E1, *Kharif* 2017; E2, *Kharif* 2018, E3, *Kharif* 2019.

Pooled analysis revealed significant variation among genotypes and environments. In GGE biplot analysis, the NIL18 was found to be the best genotype with maximum per se performance and stable across testing seasons as compared to the recurrent parent PB 1509 (Fig. 5).

### Salinity stress induced expression pattern of *OsHKT1;5* gene

*OsHKT1;5* is the putative candidate gene in the *Saltol* region which governs seedling stage salinity tolerance in FL478^33^. Therefore, the expression pattern of *OsHKT1;5* was studied in a time-series experiment under 50 mM NaCl salt stress conditions.

**Figure 6:**
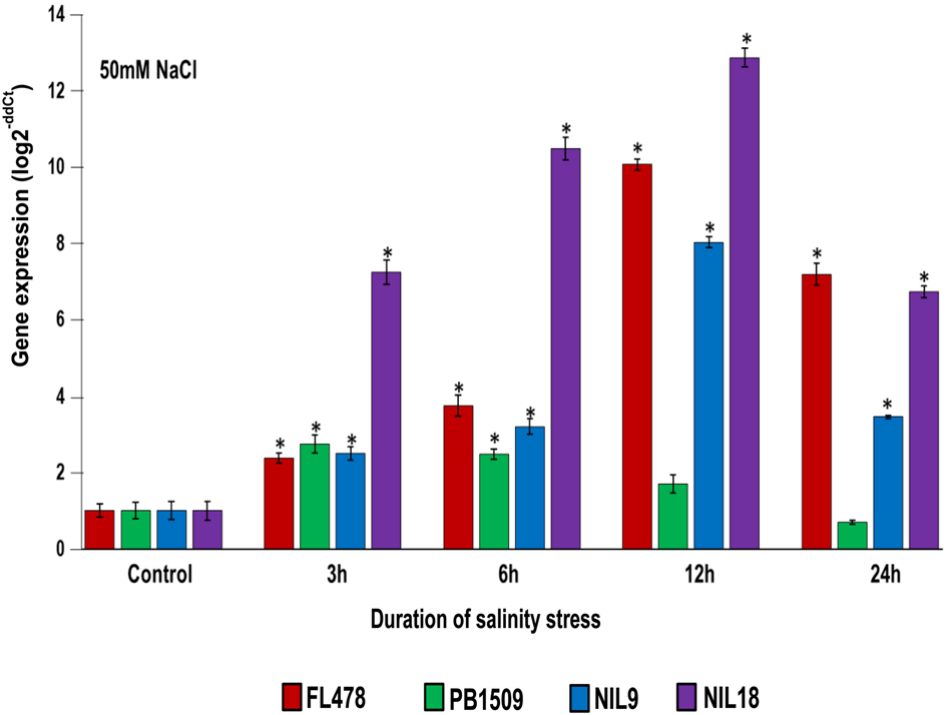
Expression profile of *OsHKT1;5* in the parental lines (PB 1509 and FL478) and PB 1509-NILs (NIL9 and NIL18) at different time intervals after exposing to 50 mM of salt stress. Bar graphs were plotted between stress hours (x-axis) and relative expression level (log 2-ddCt) (y-axis). *Student's* t-test was performed for determining the statistical significance. An asterisk (*) above the bars refers to significant differences from their respective control at *P* ≤ 0.05. The values represented are the mean of three biological replicates and standard error is shown as error bar.

Under salinity stress (50 mM) conditions, significant induction of *OsHKT1;5* was observed 3-h post stress (hps) with progressively increased expression with time and peaking to the maximum by 12 hps and further reducing after 12 h of stress. Although the trend remained the same for all the tolerant genotypes viz., FL478, NIL9, and NIL18, varied expression levels were observed among the genotypes. The maximum induction of *OsHKT1;5* was observed in NIL18 as compared to NIL9 and FL478. However, in the sensitive genotype PB 1509, the expression levels of *OsHKT1;5* was significantly lower (Fig. 6).

## Discussion

Remarkable improvement in cooked kernel length and volume expansion in high yielding Basmati rice varieties namely, Pusa Basmati 1121 and Pusa Basmati 1509 has led to their increased consumers’ preference. In addition, greater demand from domestic and international market and the consequent higher profit realization by farmers has made these cultvars as their first choice. As a result, the total area under cultivation of these varieties has increased to almost 1.3 million ha., mainly in the states of Punjab, Haryana and western Uttar Pradesh. The practise of rice cultivation in the aforesaid region is primarily based on flood irrigation using the canal and/or underground water in transplanted condition. This has led to build up of salinity, affecting rice productivity in Basmati growing areas^23^. Therefore, it is imperative to develop salt stress tolerant Basmati varieties for cultivation in these areas.

MABB is a simple and efficient methodology to rectify specific defects of an otherwise popular variety that has been successfully deployed to develop varieties resistant to bacterial blight, blast and sheath blight in rice^38–46^, particularly leading a paradigm shift in Basmati breeding^47^.

In the present paper, MABB approach which includes foreground and background selections along with phenotypic selections was used to introgress *Saltol*, a major QTL for seedling stage salinity tolerance from donor ‘FL478’ to improve ‘PB 1509’ an elite Basmati rice variety. FL478 has been extensively used to transfer *Saltol* into elite rice varieties through MABB^2, 19, 21,23,48–53^. Foreground selection followed by background and phenotypic selection has led to the precise transfer of *Saltol* QTL as well as accelerated the RPG recovery to an extent of 96.67–98.57% with three backcrosses together with the recovery of Basmati grain and cooking quality traits. The complete recovery of carrier chromosome (chromosome 1) was achieved using 18 polymorphic markers while retaining the 1 Mb region flanked by the markers AP3206f and RM10793 harbouring *Saltol* from the donor FL478 (Fig. 1). The realised RPG recovery was sufficient enough to claim the NILs genetically as close to the RP. Began with a whole genome marker coverage of approximately one marker for every 2 cM distance, the parental polymorphism survey revealed 13.3% polymorphism between parents. The polymorphic markers were distributed with an average genetic distance of 15.23 cM between two polymorphic markers. Realisation of 98.5% of the polymorphic markers has brought the actual genome similarity between the NIL and RP including the monomorphic markers to almost 99.7%. This indicates that with reasonably good background selection, we can achieve maximum recovery of RPG using microsatellite markers. Under similar situations, Ellur et al. compared the efficiency of SNPs and SSRs for estimating the RPG recovery, which did not show significant differences between the estimates^46^.

The RP, PB 1509 is well known for its superior grain, cooking quality and is highly aromatic. However, the DP, FL478, is a non-Basmati genotype with coarse grain having contrasting grain qualities such as red pericarp, bold grains and lacks aroma. Use of a genotype with inferior grain quality as the DP to transfer *Saltol* QTL to PB 1509 could potentially impair the grain and cooking quality of backcross derived lines, which sets a task for recovering the grain and cooking quality of Basmati rice in MABB. In this study, we could develop PB 1509-NILs carrying *Saltol* almost identical to the RP for agro-morphological, Basmati grain and cooking quality traits while exhibiting seedling stage salinity tolerance similar to FL478. This was possible due to stringent phenotypic selection for agronomic performance, grain and cooking quality traits for recurrent parent phenotype carried out in each of the segregating generations. The importance of phenotypic selection in augmenting background selection for maximizing the precision in developing NILs with maximum RPG and RPP recovery has also been earlier demonstrated^38,44^. The assessment of yield performance of PB 1509-NILs and RP showed that the *Saltol* QTL had set no adverse effect on yield. The NIL3 and NIL18 were found promising with significantly superior yield as compared to PB 1509 along with in-built higher level of tolerance to salt stress under field conditions (ECe of 13.9 dS/m). Although there was significant variation observed among the seasonal environments, based on the position of RP and the NILs on the biplot, NIL18 was concluded to have high per se performance and was stable across testing seasons.

Despite significant progress made in genomics assisted breeding, development of rice cultivars tolerant to salinity continues to be a major challenge due to the complex nature of *Saltol* locus. In this study, although 58 BC_3_F_2_ plants were identified to be homozygous for the *Saltol* locus, they showed varied response to salinity stress (Supplementary Table S3). This differential response to salinity stress may be attributed to the background effect or intra-QTL recombination. The background effect may vary due to unknown interactions of *Saltol* locus with the genomic regions of RP or due to the existence of other minor QTLs and their interaction with *Saltol* for rendering seedling stage salinity tolerance in the DP^2,23^. The intra-QTL recombination as a cause of differential response of *Saltol* carrying NILs may be ascertained by increasing the number of polymorphic markers in the QTL region to rule out the possibility of a double crossover between flanking markers leading to loss of QTLs yet having donor allele at flanking marker loci. Assuming this, it should have been possible to recover NILs having recurrent parent alleles at flanking peak marker loci with tolerant phenotype. However, such NILs could not be recovered as they were rejected during the selection process based on marker genotype without exposing them to salinity stress. Since the *Saltol* region spans over ~ 1.5Mbp, the possibility of intra-QTL recombination does exist, which was evident from the haplotype variation in the *Saltol* region in the rice germplasm^54^.

Salt tolerance of rice is an indicative of several components related to Na^+^ and K^+^ homeostasis. The lower Na^+^/K^+^ ratio provides protection against the toxic effects of Na^+^, hence, tolerant to salt stress^13,14^. The sensitive genotypes are known to transfer larger amounts of Na^+^ from roots to shoots which results in higher osmotic potential in their roots and thereby less water uptake from saline soil solution^55^, leading to reduced RWC. The higher concentration of Na^+^ in the shoots causes membrane injury leading to electrolyte leakage^56,57^. The significance of several fold increase in proline accumulation in the salt tolerant Pokkali and Nona Bokra under salt stress was earlier demonstrated by Ghosh et al.^58^ Therefore, RWC, MSI and proline content were also used as indices for salinity tolerance in this study. Roots play an important role in governing salinity tolerance by unloading Na^+^ from the xylem and thereby significantly reducing the amount of Na^+^ ions transported to the shoots. In the current study, the Na^+^ concentration and Na^+^/K^+^ in the roots of PB 1509-NILs carrying *Saltol* locus was significantly lower as compared to the salt susceptible RP. This may be attributed to increased accumulation of K^+^ in the roots. Therefore, these NILs were capable to maintain relatively higher turgidity with higher membrane stability and accumulated significantly higher amount of low-molecular weight solutes such as proline. Therefore, the cation balance through modulation of Na^+^ transport and accumulation of low-molecular weight solutes could be the possible mechanisms of action of *Saltol* imparting seedling stage salinity tolerance.

In a population generated from a cross of IR29/Pokkali, *Saltol*, a QTL governing seedling stage salinity tolerance was mapped on chromosome 1 and was delimited to 10.7–12.2 mb^14^. In this study, it was reported that *Saltol* could explain 43% of variation for Na^+^/K^+^ ratio than any other QTLs. Being mapped very close to *Saltol*, the *shoot* *K*^+^ *concentration 1* *(SKC1)* a Na^+^/K^+^ transporter has been hypothesized to be the putative candidate gene governing seedling stage salinity tolerance. *SKC1* was later identified to be a member of the high-affinity K^+^ transporter 1 (*HKT1*) family, known as *OsHKT1;5* and coded for a xylem-expressed Na^+^ transporter that regulates cation homeostasis under salt stress in rice^17,18^. We could observe a significant up-regulation of *OsHKT1*;5 among the salt tolerant NILs as compared to the salt susceptible PB 1509 demonstrating the role of *OsHKT1*;5 in governing salinity tolerance through Na^+^ homeostasis under salt stress conditions. The results corroborated with the Na^+^ concentration in the shoots of NILs and RP under salt stress conditions^23^. Further, upon induction of salt-stress, the expression levels of *OsHKT1;5* was progressively increased from 3 to 12 hps and decreased gradually thereafter in the PB 1509-NILs, denoting successful introgression of *Saltol *QTL into the NILs.

Despite being implicated as the major gene of the *Saltol* locus, the role of *OsHKT1;5* remained inconclusive in imparting salt tolerance in rice for a long time, for the absence of a knockdown mutant. Kobayashi et al*.*^59^ demonstrated that loss of function of *OsHKT1;5* in two DNA insertional mutant, could trigger accumulation of Na^+^ in shoots, leading to salt injury. They found that *OsHKT1;5* remains localised in cells adjacent to xylem, and involves in Na^+^ unloading from xylem, while in basal nodes, it accumulates in phloem to prevent delivery of Na^+^ to young leaves. Recently, functional variation has been reported in *OsHKT1;5* in imparting salt tolerance associated with two amino acid substitutions in Pokkali^60^. Such variations are not expected in the present case, as all of the NILs have inherited the gene from the common donor, FL478. In this study, we have not observed any post stress induction of *OsHKT1;5* in the sensitive genotype PB 1509, indicating that the *OsHKT1;5* expression is associated with salt stress tolerance via regulating Na^+^/K^+^ homeostasis as found in other studies also^60^. Further, SNP variation in *OsHKT1;5* has been identified between salt tolerant and susceptible rice genotypes, evidencing that *OsHKT1;5* is the major gene governing salt tolerance imparted by *Saltol* QTL^61^. Notwithstanding, *Saltol* region is quite long, and harbours loci that modify the expression of salt tolerance, that has puzzled the breeders who tried to introgress this QTL into different genetic backgrounds. For instance, the increased salt tolerance of FL478 relative to Pokkali was reported due to the presence of an IR 29 fragment in the vicinity of *Saltol* QTL^33^. While transferring the Pokkali QTL from FL478, it is likely that, this additional fragment could be lost due to genetic recombination. The fact that IR29 fragment has no direct effect on salt tolerance, can therefore result in lower salt tolerance among such recombinants. In this study, NIL9 and NIL18 had differences in the magnitude of salt tolerance expression, as well as had different level of RPG recovery for the donor parent fragment, indicates such a possibility. Analogous difference in expression levels between two NILs may be attributed to any factor such as background interaction, intra- or extra-QTL recombination. Therefore, further studies are required to associate the role of DP introgressions on the magnitude of expression of *OsHKT1*;5 under salt stress conditions.

In all, the present study has led to the development of improved PB 1509 with tolerance to salinity stress through MABB. The lines developed in the current study will be boon to the Basmati farmers to realize the potential yield in the salt affected soils of the north-western part of the Indian sub-continent.

## Materials and methods

### Plant materials and crossing scheme

**Figure 7:**
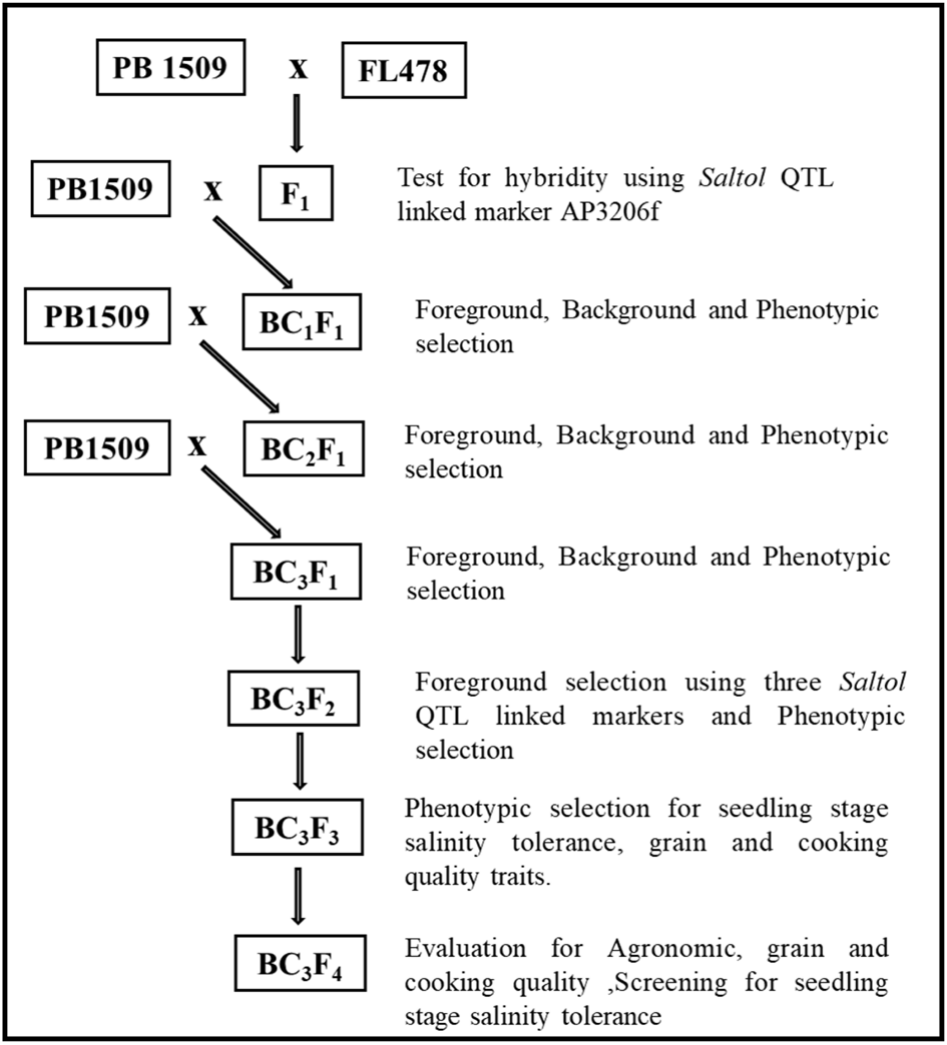
Marker assisted backcross breeding scheme used for introgression of *Saltol* in RP-PB 1509.

PB 1509 was used as the RP and FL478 as the DP for *Saltol* in MABB (Fig. 7). RP and DP were first screened for seedling stage salinity tolerance at 120 mM (mM NaCl concentration in hydroponic solution (EC of 13.9 dS/m) to validate their reaction for salinity. PB 1509 was crossed with FL478 to obtain F_1_ seeds and hybridity of the F_1_ plants was confirmed using the *Saltol* QTL linked SSR markers viz*.* AP3206f, RM3412b, and RM10793. The plant selected in F_1_ was designated as Pusa1960. The selected F_1_ plant was backcrossed with PB 1509 to generate BC_1_F_1_ seeds. The BC_1_F_1_ plants were subjected to foreground and background selection. Background selection in every generation was coupled with a comprehensive phenotypic selection for agronomic, grain and cooking quality traits to accelerate the recovery of recurrent parent phenome (RPP). One BC_1_F_1_ plant with the highest RPG recovery and phenotypic similarity to the RP was backcrossed to generate the BC_2_F_1_ seeds. In the BC_2_F_1_ and BC_3_F_1_ generations, the same strategy was followed to identify plants with desired allelic combination at the three foreground markers in the target region along with maximum recovery for RPG. The superior BC_3_F_1_ plants were advanced to BC_3_F_2_ generation and subjected to foreground selection using all the three foreground markers to identify homozygous plants. Further, the selected BC_3_F_2_ plants were advanced to raise BC_3_F_3_ families, which were screened for seedling stage salinity tolerance. The plants showing the highest level of salinity tolerance were advanced to BC_3_F_4_ generation.

### Molecular marker analysis

Total genomic DNA was isolated from leaf tissue of test lines using Cetyl Trimethyl Ammonium Bromide (CTAB) method^25^. Polymerase chain reaction (PCR) was performed in a thermal cycler (Applied Biosystem Veriti, California, USA) using a total reaction mix of 10 μl by adding 25–30 ng of genomic DNA. 5 pmol of each of the two primers (synthesized from Sigma-Aldrich Inc., St. Louis, MO, USA), 0.2 mM dNTPs, 1.5 mM MgCl_2_, (MBI, Fermentas, Vilnius, Lithuania) and 0.5 U of Taq polymerase (Bangalore Genei, Bangalore, India). PCR amplification was performed by one cycle of denaturation at 95 °C for 4 min, followed by 35 cycles at 95 °C for 40 s, 55 °C for 40 s and 72 °C for 1 min, with a final extension of 72 °C for 10 min. The PCR amplified products were resolved on 3.5% Metaphor Agarose gel containing 0.1 mg/mL of ethidium bromide (Amresco, Solon, OH, USA) along with a DNA size standard 50 bp ladder (MBI, Fermentas, Vilnius, Lithuania) and visualized on ultraviolet trans-illuminator (Gel Doc XR + Gel Documentation system, Bio-Rad Laboratories Inc.,U.S.A).

### Foreground and background selection

For foreground selection, 30 markers linked to *Saltol* locus comprising of 26 SSR markers and 4 STS markers falling within the region between 10.8 mb and 15.8 mb on chromosome 1^16^ were used in parental polymorphism survey. Three markers tightly linked to *Saltol* locus namely AP3206f, RM3412b and RM10793 were found to be polymorphic and were used in the foreground selection. The details on primers used in foreground selection are presented in Supplementary Table S1. For Background selection, a total of 786 genome-wide SSR markers sourced from the rice marker data base at Gramene (https://www.gramene.org) were used for the polymorphism survey between RP and DP and 105 markers were identified to be polymorphic, which were used for background selection to estimate the RPG recovery (Supplementary Table S2).

### Screening for seedling stage salt tolerance

The NILs along with the parents and susceptible check IR29 were evaluated under hydroponics in the glasshouse of National Phytotron Facility at ICAR-IARI, New Delhi and under field conditions (micro-plot) at ICAR-IARI, New Delhi, India.

Under hydroponics conditions, the screening was conducted in completely randomized design (CRD) with three replications. The data was recorded on ten plants from each of the genotypes in every replication. The seeds were surface sterilized with 5% Sodium hypochlorite solution for 30 min and rinsed with distilled water several times. Sterilized seeds were then placed for germination with moistened filter paper in Petri dishes and kept for 72 h at 30 °C. The seedlings were grown by placing a healthy pre-germinated seed in the hole punched on a polystyrene foam sheet that contained a 16 × 10 matrix of holes. The polystyrene sheet was made to float in the nutrient solution taken in a plastic crate. The sheet was lined with a nylon wire mesh on the bottom side stitched intact to prevent seeds from falling into the solution^26^. The container was filled with 12 L of Yoshida nutrient solution^27^. Fourteen days after germination, the container was added with saline solution having 60 mM NaCl (EC of 6.9 dS/m) imposing 50% of the proposed stress level and after 3 days, salinity stress was raised to 120 mM (EC of 13.9 dS/m), which was maintained until final phenotypic scoring. The container was refilled with fresh nutrient solution maintaining the required salinity level at every 4 days’ interval. The genotypes were visually scored using modified standard evaluation system (SES) for rice for salt stress symptoms^28^, with scores ranging from 1 (highly tolerant) to 9 (highly sensitive) on 16^th^ day after the first salinization. Further, the leaf samples were collected for estimation of relative water content (RWC), membrane stability index (MSI) and proline content.

For field evaluation, the seeds of PB 1509-NILs and their parental lines along with susceptible check IR29 were evaluated in Randomized Complete Block Design (RCBD) with three replications in micro-plots. The plot was maintained with the pH_2_ ∼ 7.8 (pH_2_ measured by 1 part soil and 2 parts distilled water and here in after will be denoted as pH). Eight days after germination, the saline solution having EC of 6 dS/m was applied and 3 days later the salt stress level was increased by applying the saline solution with EC of 13.9 dS/m. The salt stress level was maintained by irrigating the plants with saline water (7NaCl: 1Na_2_SO_4_: 2CaCl_2_ on equivalent basis). The NILs were visually scored after complete death of susceptible parent and susceptible check IR29. The shoot and root samples were collected and rinsed with distilled water for three times before dried in a hot air oven at 80 °C for 3 days. Dried samples of shoot and root were used for assessment of Na^+^ and K^+^ ion concentration.

### Determination of leaf RWC

RWC was estimated following the protocol of Barrs and Weatherley^29^, wherein, fully expanded third leaf from the top from unstressed and stressed plants were used. To minimize water loss, the leaf samples were placed in a plastic bag kept on an ice pack (around 4 °C) and shifted to the laboratory directly. Leaf fresh weight (FW) was recorded and then hydrated to full turgidity by placing leaf in 50 mL capped vials filled with 25 mL of deionized water, for 6 h (h) at 4 °C. After 6 h, surface of leaves were wiped with lint free filter paper and turgid weight (TW) of the leaves were recorded. Samples were then kept for drying in hot air oven at 80 °C for 48 h and dry weight was recorded. RWC was computed as follows:

$${\text{RWC}} = \, \left[ {\left( {{\text{FW }} - {\text{ DW}}} \right)/\left( {{\text{TW }} - {\text{ DW}}} \right)} \right] \, \times { 1}00.$$

### Evaluation of MSI

MSI was estimated by measuring the electrolyte leakage into deionized water from fresh leaf tissues using the method described by Sairam et al.^30^. Samples collected from unstressed and stressed plants were rinsed several times with deionized water to remove electrolytes from the surface of the leaf. About 100 mg leaf was cut into very small pieces (0.5 cm) and placed in test tubes containing 25 mL of distilled deionized water. Test tubes were incubated at constant temperature of 40 °C in water for 2 h. Electrical conductivity (EC_1_) of samples was recorded after 2 h using an electrical conductivity meter (Model CMK-731; Century Instruments). After the first measurement, the test tubes were again incubated at 100 °C in a boiling water bath for 15 min to kill the tissues and discharge electrolytes. Further, the samples were cooled to 25 °C and electrical conductivity (EC_2_) was measured again. These two measurements were recorded individually from both the unstressed and stressed condition for all the samples. The MSI was calculated as:

$${\text{MSI}}\,\left( \% \right) \, = \, \left( {{1} - {\text{ EC}}_{{1}} {\text{/EC}}_{{2}} } \right) \times { 1}00.$$

### Estimation of proline

Proline was estimated following Bates et al.^31^. Leaf material was ground to a fine powder in liquid nitrogen using a pestle and mortar. 500 mg of fine powder was homogenized in 10 mL of 3% aqueous sulfosalicylic acid (w/v) and the homogenate sieved through Whatman #2 filter paper. Two mL of the filtrate was reacted with 2 mL of glacial acetic acid and 2 mL of acid-ninhydrin reagents in a test tube and placed in a boiling water bath for one hour and tubes were immediately placed in ice to terminate the reaction. To this reaction mixture, 4 mL toluene was added and mixed vigorously using a vortex for 20–30 s. The yellow colour chromophore containing toluene was aspirated from the aqueous phase, warmed to room temperature and the absorbance was read using a spectrophotometer (Thermo Fisher Scientific, USA) at 520 nm using toluene as a blank. The proline concentration was determined from a standard graph and calculated in μ moles proline/g of fresh weight material.

### Estimation of Na^+^ and K^+^ content in shoots and roots

For estimation of the Na^+^ and K^+^ ion concentrations in shoots and roots of the salt-stressed and unstressed NILs and parents, the oven dried plant materials were ground to a fine powder. About 500 mg of the powder was added into a test tube and mixed with 15 mL of diacid digestion mixture (HNO_3_ and HClO_4_, 10:3). The digest was cooled and transferred to a 50 mL volumetric flask and volume was made up to 50 mL^27,32^. The mixture was filtered with Whatman number 42 filter paper and concentration Na^+^ and K^+^ were estimated using a Systronics Flame Photometer 128 (SYSTRONICS India).

### Agronomic performance and grain quality assessment

Agronomic performance of the 20 NILs and parents (PB 1509 and FL478) was evaluated in RCBD with three replications during *Kharif* 2017, 2018, and 2019 and the trial was maintained following recommended agronomic practices. From each replication, data on days to 50% flowering (DFF), plant height (PH), number of effective tillers per plant (NT), panicle length (PL), spikelet fertility (SF), and thousand grain weight (TW) were recorded on five plants. The plot yield was recorded for each of the replication in kilogram per hectare (kg/ha). The grain and cooking quality traits such as hulling recovery (HUL), milling recovery (MIL), head rice recovery (HRR), kernel length before cooking (KLBC), kernel breadth before cooking (KBBC), kernel breadth after cooking (KBAC), kernel length after cooking (KLAC), kernel elongation ratio (ER), alkali spreading value (ASV), and aroma were recorded as described in Babu et al^23^.

### Expression profiling of *OsHKT1;5* gene

The expression of *OsHKT1;5* gene was examined in two NILs namely, Pusa 1960-3-25-7-205 (NIL9) and Pusa 1960-3-25-7-423 (NIL18) along with their parents PB 1509 and FL478. These genotypes were grown in Yoshida solution for 21 days in three replications. Further, they were exposed to salinity level at 50 mM (moderate stress) NaCl in two separate experiments together with unstressed control. Leaf tissues were collected at 0, 3, 12, and 24 h after imposing stress. Total RNA was isolated from each of the genotypes using Nucleo Spin RNA kit (MACHERY-NAGEL, Germany) following the manufacturer’s protocol, and was quantified using NanoDrop ND1000 spectrophotometer (Thermo Fisher Scientific, USA). cDNA was synthesized using the SuperScript VILO cDNA Synthesis Kit (Invitrogen, Thermo Fisher Scientific, USA). The expression analysis of *OsHKT1:5* (F-TTCATGGCGGTCAACTCGA and R-TTTGCTGGTGTTTGTCTTGGA)^33^ was conducted using qRT-PCR with 18s rRNA (F-TGATAACTCGACGGATCGC, R-CTTGGATGTGGTAGCCGTTT)^34^ was used as an endogenous control. All the reactions were performed in a QuantStudio 12 K flex real time PCR (Applied Biosystem, Thermo Fisher Scientific, USA) using Power SYBR Green master mix (Applied Biosystem, Thermo Fisher Scientific, USA) with three biological and three technical replicates for each of the genotypes. The amplification were analyzed using Applied Biosystems QuantStudio 12 K Flex system software v1.2.2 (www.thermofisher.com/in/en/home/global/forms/quantstudio-12k-flex-software-download.html).

### Statistical analyses

The agro-morphological data were analyzed for standard statistical tests using the software package CropStat 7.2^35^. Relative quantitative expression of *O*s*HKT1;5* gene was calculated based on log fold change (ΔΔCT method) compared with respective controls^36^ and Student’s t-test was performed for statistical significance differences from their respective control at *P* ≤ 0.05 using SAS software Version 9.3^37,38^.

## Supplementary information

Supplementary Figures.

Supplementary Tables.
