# Supplementary material for: Marker aided introgression of ‘Saltol’, a major QTL for seedling stage salinity tolerance into an elite Basmati rice variety ‘Pusa Basmati 1509’
Source: Sci Rep. 2020 Sep 4;10:13877. doi: 10.1038/s41598-020-70664-0 (PMC7474085; doi:10.1038/s41598-020-70664-0)
Supplement: Supplementary file 1 — Supplementary Figures. [file 41598_2020_70664_MOESM1_ESM.docx]

**Marker aided introgression of ‘*Saltol’*, a major QTL for seedling stage salinity tolerance into an elite Basmati rice variety ‘Pusa Basmati 1509’**

Ashutosh Kumar Yadav^1,2^, Aruna Kumar^2^, Nitasha Grover^1^, Ranjith Kumar Ellur^1^, Gopala Krishnan S.^1^, Haritha Bollinedi^1^, Prolay Kumar Bhowmick^1^, K.K. Vinod^3^ , Nagarajan M^3^ , S.L. Krishnamurthy^4^ and Ashok Kumar Singh^*1,^


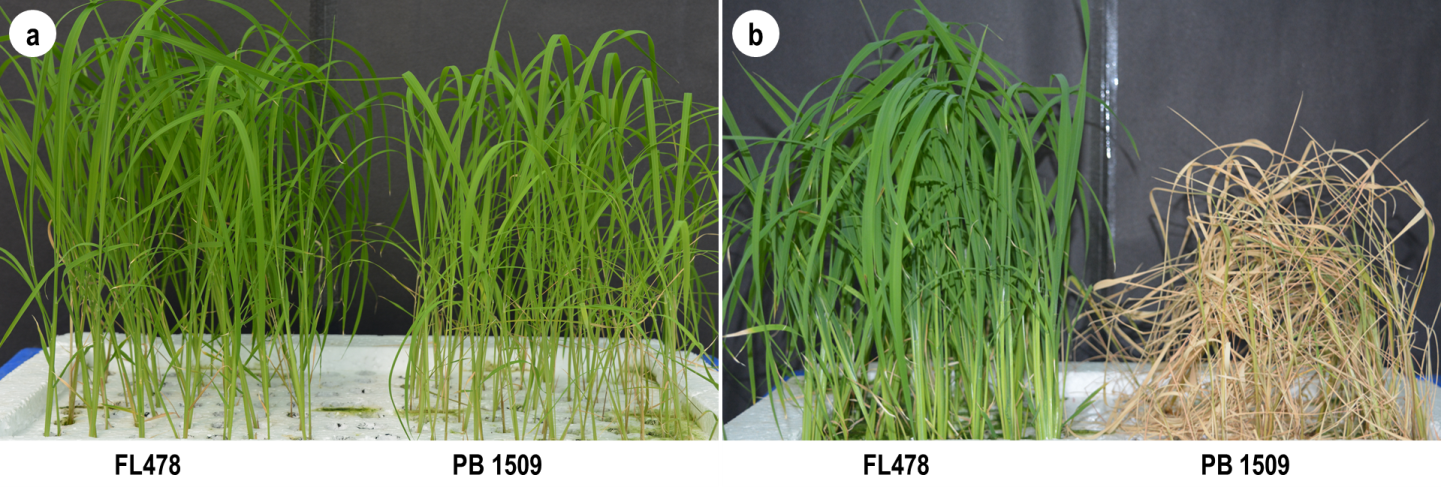


**Supplementary Figure S1.** Phenotypic evaluation of RP (PB 1509) and DP (FL478) for reaction under salt stress at the seedling stage at 120mM NaCl (EC of 13.9 dSm^−^), (a) un-stressed, (b) salt-stressed condition.


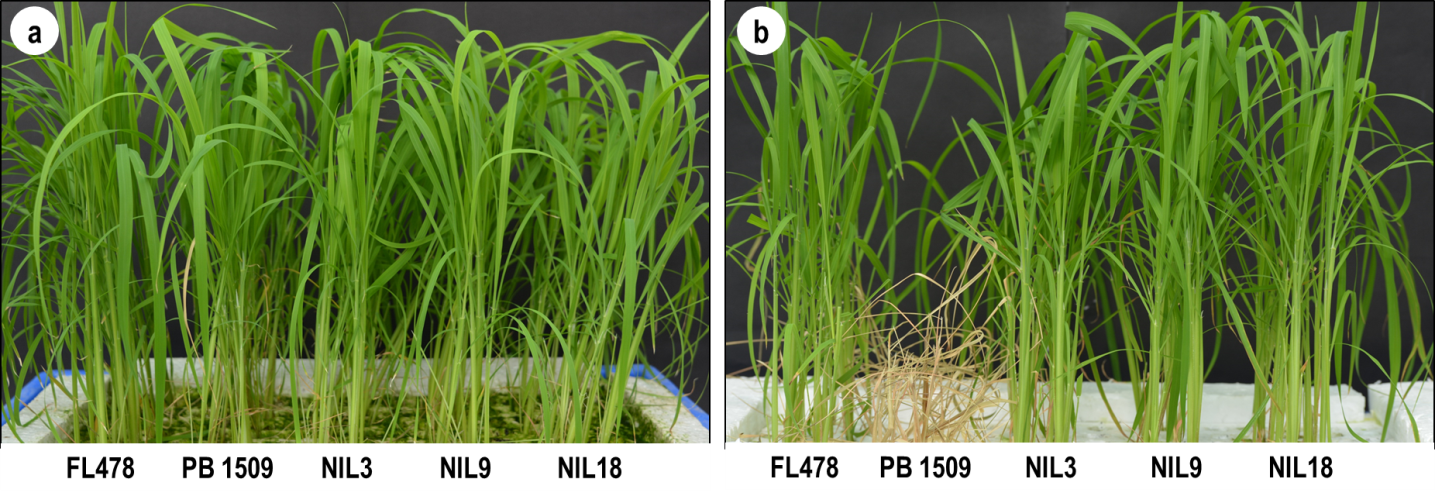


**Supplementary Figure S2.** Representative picture of Screening of *Saltol*-introgressed PB 1509-NILs (BC_3_F_3_) along with RP (PB 1509) and DP (FL478) for seedling stage salinity tolerance under hydroponic solution at 120mM NaCl (EC of 13.9 dSm^−1^), (a) un-stressed, (b) salt-stressed condition.


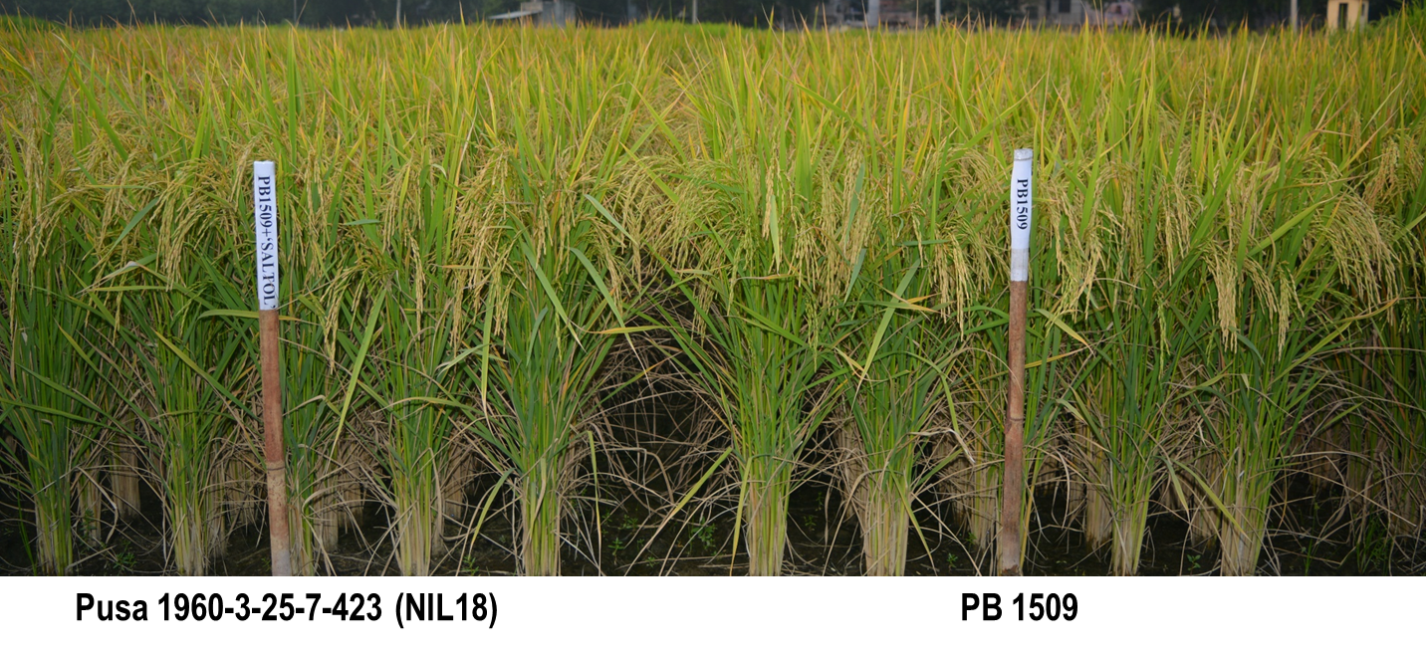


**Supplementary Figure S3.** Field view of RP-PB 1509 and its NIL Pusa 1960-3-25-7-423 (NIL18) carrying ‘*Saltol’.*


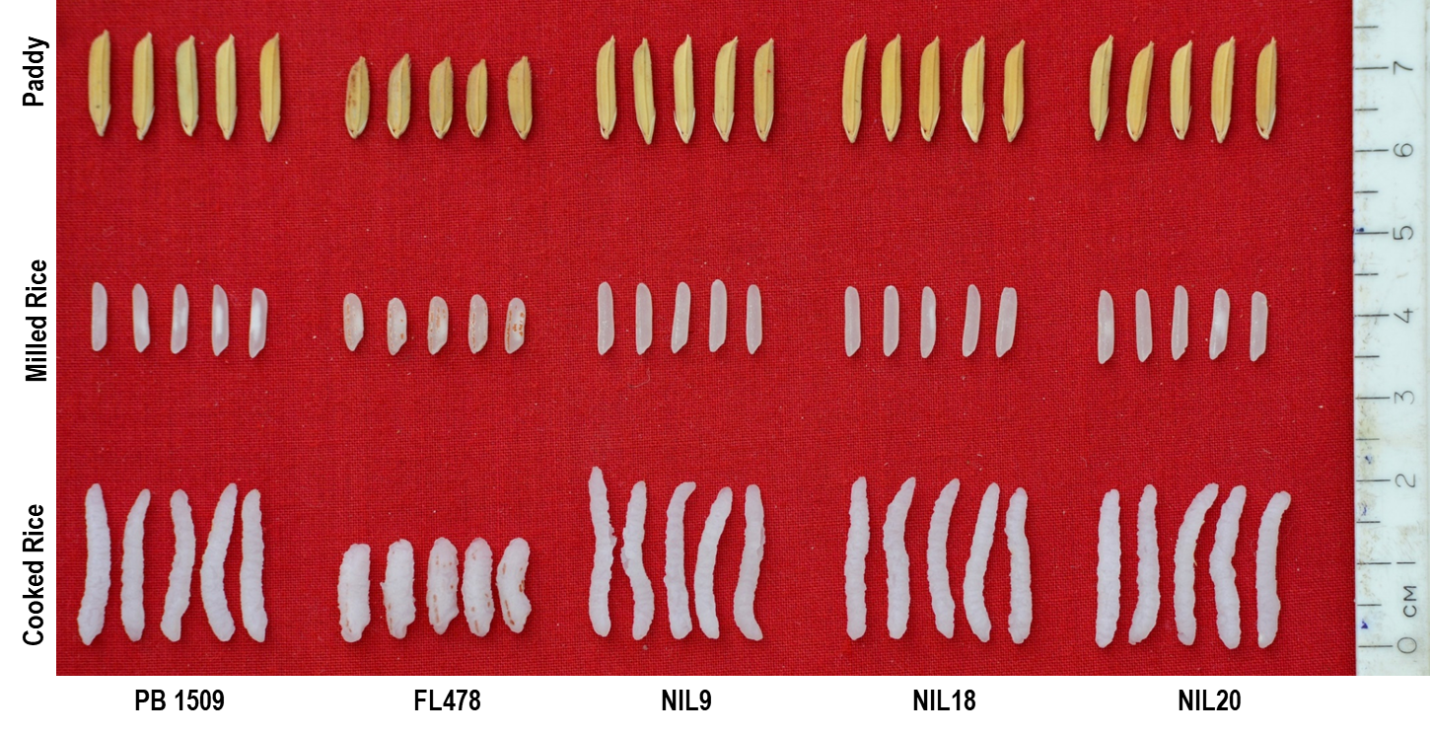


**Supplementary Figure S4**. Grain and cooking quality of parental lines and PB 1509-NILs carrying Saltol QTL.
