# Supplementary material for: Marker aided introgression of ‘Saltol’, a major QTL for seedling stage salinity tolerance into an elite Basmati rice variety ‘Pusa Basmati 1509’
Source: Sci Rep. 2020 Sep 4;10:13877. doi: 10.1038/s41598-020-70664-0 (PMC7474085; doi:10.1038/s41598-020-70664-0)
Supplement: Supplementary file 2 — Supplementary Tables. [file 41598_2020_70664_MOESM2_ESM.docx]

**Marker aided introgression of ‘*Saltol’*, a major QTL for seedling stage salinity tolerance into an elite Basmati rice variety ‘Pusa Basmati 1509’**

Ashutosh Kumar Yadav^1,2^, Aruna Kumar^2^, Nitasha Grover^1^, Ranjith Kumar Ellur^1^, S. Gopala Krishnan^1^, Haritha Bollinedi^1^, Prolay Kumar Bhowmick^1^, K. K. Vinod^1^, M. Nagarajan^3^, S. L. Krishnamurthy^4^ and Ashok Kumar Singh^1*^

**Supplementary Table S1.** List of SSR markers linked to the *Saltol* QTL which were used in foreground selection

| **Markers** | **Primer Sequences** | **Tm** | **PB 1509 Allele** | **FL478 Allele** | **Position (Mb)** |
| --- | --- | --- | --- | --- | --- |
| AP3206f | F: GCAAGAATTAATCCATGTGAAAGA | 55℃ | 325 bp | 350 bp | 11.2 |
|  | R: ATGCTCTGGCTCCCTCAAG |  |  |  |  |
| RM3412b | F: TCATGATGGATCTCTGAGGTG | 55℃ | 130 bp | 150 bp | 11.5 |
|  | R: GGGAGGATGCACTAATCTTTC |  |  |  |  |
| RM10793 | F: GACTTGCCAACTCCTTCAATTCG | 55℃ | 180 bp | 130 bp | 12.5 |
|  | R: TCGTCGAGTAGCTTCCCTCTCTACC |  |  |  |  |

**Supplementary Table S2.** Details of SSR markers used for parental polymorphism survey

| **Markers used for** | **Total No. of Markers**  **surveyed** | **Total No. of Polymorphic**  **markers** | **Polymorphic**  **Markers used** | **Polymorphism**  **(%)** |
| --- | --- | --- | --- | --- |
| **Foreground Selection** | 30 | 3 | 3 | 10.00 |
| **Background Selection*** | 786 | 105 | 105 | 13.35 |
| **Chromosome 1** | 96 | 18 | 18 | 18.75 |

**∗ includes markers on chromosome 1.**

**Supplementary Table S3.** The reaction of BC_3_F_3_ families homozygous for *Saltol* QTL under salinity stress conditions (120mM NaCl) EC of 13.9 dS/m.

| **S.No** | **Plant ID** | **Foreground Selection** | | | **STS** | **Salinity reaction** |
| --- | --- | --- | --- | --- | --- | --- |
|  |  | **AP3206f** | **RM3412b** | **RM10793** |  |  |
| 1 | Pusa 1960-3-25-7-1 | **++** | **++** | **++** | 5 | M |
| 2 | Pusa 1960-3-25-7-3 | **++** | **++** | **++** | 1 | HT |
| 3 | Pusa 1960-3-25-7-8 | **++** | **++** | **++** | 1 | HT |
| 4 | Pusa 1960-3-25-7-18 | **++** | **++** | **++** | 5 | M |
| 5 | Pusa 1960-3-25-7-25 | **++** | **++** | **++** | 1 | HT |
| 6 | Pusa 1960-3-25-7-34 | **++** | **++** | **++** | 6 | M |
| 7 | Pusa 1960-3-25-7-38 | **++** | **++** | **++** | 1 | HT |
| 8 | Pusa 1960-3-25-7-42 | **++** | **++** | **++** | 1 | HT |
| 9 | Pusa 1960-3-25-7-48 | **++** | **++** | **++** | 5 | M |
| 10 | Pusa 1960-3-25-7-50 | **++** | **++** | **++** | 1 | HT |
| 11 | Pusa 1960-3-25-7-55 | **++** | **++** | **++** | 1 | HT |
| 12 | Pusa 1960-3-25-7-66 | **++** | **++** | **++** | 6 | M |
| 13 | Pusa 1960-3-25-7-72 | **++** | **++** | **++** | 6 | M |
| 14 | Pusa 1960-3-25-7-79 | **++** | **++** | **++** | 6 | M |
| 15 | Pusa 1960-3-25-7-88 | **++** | **++** | **++** | 5 | M |
| 16 | Pusa 1960-3-25-7-92 | **++** | **++** | **++** | 5 | M |
| 17 | Pusa 1960-3-25-7-100 | **++** | **++** | **++** | 5 | M |
| 18 | Pusa 1960-3-25-7-104 | **++** | **++** | **++** | 5 | M |
| 19 | Pusa 1960-3-25-7-110 | **++** | **++** | **++** | 5 | M |
| 20 | Pusa 1960-3-25-7-118 | **++** | **++** | **++** | 5 | M |
| 21 | Pusa 1960-3-25-7-122 | **++** | **++** | **++** | 1 | HT |
| 22 | Pusa 1960-3-25-7-128 | **++** | **++** | **++** | 1 | HT |
| 23 | Pusa 1960-3-25-7-136 | **++** | **++** | **++** | 6 | M |
| 24 | Pusa 1960-3-25-7-156 | **++** | **++** | **++** | 1 | HT |
| 25 | Pusa 1960-3-25-7-162 | **++** | **++** | **++** | 1 | HT |
| 26 | Pusa 1960-3-25-7-198 | **++** | **++** | **++** | 1 | HT |
| 27 | Pusa 1960-3-25-7-205 | **++** | **++** | **++** | 1 | HT |
| 28 | Pusa 1960-3-25-7-221 | **++** | **++** | **++** | 1 | HT |
| 29 | Pusa 1960-3-25-7-224 | **++** | **++** | **++** | 5 | M |
| 30 | Pusa 1960-3-25-7-234 | **++** | **++** | **++** | 1 | HT |
| 31 | Pusa 1960-3-25-7-246 | **++** | **++** | **++** | 1 | HT |
| 32 | Pusa 1960-3-25-7-264 | **++** | **++** | **++** | 1 | HT |
| 33 | Pusa 1960-3-25-7-266 | **++** | **++** | **++** | 6 | M |
| 34 | Pusa 1960-3-25-7-284 | **++** | **++** | **++** | 5 | M |
| 35 | Pusa 1960-3-25-7-286 | **++** | **++** | **++** | 5 | M |
| 36 | Pusa 1960-3-25-7-290 | **++** | **++** | **++** | 1 | HT |
| 37 | Pusa 1960-3-25-7-291 | **++** | **++** | **++** | 5 | M |
| 38 | Pusa 1960-3-25-7-296 | **++** | **++** | **++** | 1 | HT |
| 39 | Pusa 1960-3-25-7-300 | **++** | **++** | **++** | 5 | M |
| 40 | Pusa 1960-3-25-7-302 | **++** | **++** | **++** | 6 | M |
| 41 | Pusa 1960-3-25-7-312 | **++** | **++** | **++** | 5 | M |
| 42 | Pusa 1960-3-25-7-338 | **++** | **++** | **++** | 6 | M |
| 43 | Pusa 1960-3-25-7-344 | **++** | **++** | **++** | 5 | M |
| 44 | Pusa 1960-3-25-7-352 | **++** | **++** | **++** | 5 | M |
| 45 | Pusa 1960-3-25-7-358 | **++** | **++** | **++** | 5 | M |
| 46 | Pusa 1960-3-25-7-366 | **++** | **++** | **++** | 1 | HT |
| 47 | Pusa 1960-3-25-7-379 | **++** | **++** | **++** | 5 | M |
| 48 | Pusa 1960-3-25-7-380 | **++** | **++** | **++** | 1 | HT |
| 49 | Pusa 1960-3-25-7-388 | **++** | **++** | **++** | 5 | M |
| 50 | Pusa 1960-3-25-7-392 | **++** | **++** | **++** | 5 | M |
| 51 | Pusa 1960-3-25-7-400 | **++** | **++** | **++** | 1 | HT |
| 52 | Pusa 1960-3-25-7-404 | **++** | **++** | **++** | 1 | HT |
| 53 | Pusa 1960-3-25-7-416 | **++** | **++** | **++** | 1 | HT |
| 54 | Pusa 1960-3-25-7-423 | **++** | **++** | **++** | 1 | HT |
| 55 | Pusa 1960-3-25-7-444 | **++** | **++** | **++** | 5 | M |
| 56 | Pusa 1960-3-25-7-450 | **++** | **++** | **++** | 1 | HT |
| 57 | Pusa 1960-3-25-7-452 | **++** | **++** | **++** | 1 | HT |
| 58 | Pusa 1960-3-25-7-458 | **++** | **++** | **++** | 1 | HT |
| 59 | PB 1509 | **--** | **--** | **--** | 9 | HS |
| 60 | FL478 | **++** | **++** | **++** | 1 | HT |

**HT: Highly Tolerant, M: Moderate, HS: Highly Susceptible**

**Supplementary Table S4**: Effect of salt on physiological traits on parental lines and PB 1509-NILs carrying *Saltol* QTL at seeding stage at 120mM salt stress. MSI, Membrane stability Index; RWC, Leaf relative water content; Pro, Proline content in µmoles proline/g of fresh wt; CD, Critical difference.

| **NILs** | **RWC %** | | **MSI %** | | **Proline**  (µmoles/g of fresh wt) | |
| --- | --- | --- | --- | --- | --- | --- |
|  | **Un-stressed** | **Salt Stressed** | **Un-stressed** | **Salt Stressed** | **Un-stressed** | **Salt Stressed** |
| NIL1 | 86.73±0.23 | 70.05±1.63 | 80.35±2.95 | 63.24±2.47 | 7.39±0.27 | 15.47±0.84 |
| NIL2 | 83.16±0.55 | 74.62±1.03 | 82.06±1.95 | 75.18±3.87 | 7.87±0.32 | 22.21±0.78 |
| NIL3 | 85.51±1.83 | 69.84±0.71 | 81.42±2.51 | 68.25±1.31 | 8.46±0.58 | 18.89±0.80 |
| NIL4 | 84.10±0.39 | 68.41±1.93 | 80.51±1.34 | 65.83±1.63 | 8.16±0.69 | 17.44±1.22 |
| NIL5 | 80.31±0.91 | 72.09±1.87 | 87.22±0.78 | 67.83±1.27 | 7.25±0.59 | 20.55±1.00 |
| NIL6 | 81.09±0.55 | 74.17±0.27 | 86.57±4.15 | 71.16±1.66 | 7.42±1.42 | 23.07±1.03 |
| NIL7 | 82.63±0.41 | 74.61±1.01 | 88.63±1.66 | 74.27±1.91 | 8.02±1.15 | 18.19±1.22 |
| NIL8 | 82.61±1.12 | 72.75±2.80 | 82.48±5.22 | 71.85±2.67 | 7.34±0.49 | 20.59±1.95 |
| NIL9 | 80.73±1.38 | 75.99±0.94 | 82.12±2.50 | 78.25±2.29 | 8.76±0.51 | 25.41±0.74 |
| NIL10 | 79.46±0.90 | 74.95±1.93 | 79.28±3.07 | 64.84±1.48 | 8.08±0.95 | 15.82±0.30 |
| NIL11 | 81.24±1.07 | 76.26±1.33 | 91.85±4.97 | 65.83±1.36 | 6.66±0.48 | 22.27±1.18 |
| NIL12 | 81.32±1.87 | 73.99±1.89 | 89.05±3.09 | 65.42±1.66 | 7.4±1.41 | 18.68±0.88 |
| NIL13 | 78.26±1.63 | 69.92±0.42 | 85.61±1.48 | 69.96±1.57 | 7.73±1.18 | 16.27±0.10 |
| NIL14 | 82.14±2.33 | 76.82±0.84 | 80.31±4.74 | 63.41±1.87 | 7.56±0.34 | 24.24±0.82 |
| NIL15 | 78.80±2.24 | 70.99±1.14 | 81.69±3.91 | 64.19±2.40 | 7.17±0.27 | 15.27±0.23 |
| NIL16 | 83.80±1.24 | 74.36±1.36 | 81.28±1.54 | 64.54±0.39 | 7.37±1.5 | 22.79±0.90 |
| NIL17 | 81.09±0.55 | 76.44±0.62 | 80.7±4.17 | 77.20±2.37 | 7.34±0.90 | 18.13±0.58 |
| NIL18 | 84.61±2.90 | 78.98±1.26 | 80.62±1.79 | 79.25±1.95 | 7.03±0.23 | 25.51±1.53 |
| NIL19 | 82.31±0.69 | 75.48±1.45 | 84.52±1.62 | 66.11±2.53 | 6.44±0.70 | 20.25±1.54 |
| NIL20 | 78.44±2.02 | 74.40±0.53 | 83.33±2.53 | 61.84±0.77 | 7.43±1.44 | 16.13±0.52 |
| PB 1509 | 84.47±0.58 | 42.46±1.87 | 81.85±2.3 | 49.13±0.67 | 7.54±0.49 | 10.26±0.90 |
| FL478 | 91.80±1.06 | 79.21±0.42 | 91.61±3.69 | 77.08±1.12 | 8.41±0.4 | 24.31±1.77 |
| **CD(0.05)** | **2.90** | **2.96** | **6.56** | **4.12** | **1.23** | **2.24** |

| **Un-stressed** | | | | | | | **Salt Stressed** | | | | | |
| --- | --- | --- | --- | --- | --- | --- | --- | --- | --- | --- | --- | --- |
| **NILs** | **Shoot** | | | **Root** | | | **Shoot** | | | **Root** | | |
|  | **Na^+^** | **K^+^** | **Na^+^/K^+^** | **Na^+^** | **K^+^** | **Na^+^/K^+^** | **Na^+^** | **K^+^** | **Na^+^/K^+^** | **Na^+^** | **K^+^** | **Na^+^/K^+^** |
| NIL1 | 0.19 ±0.01 | 0.89 ±0.04 | 0.22 ±0.03 | 0.32 ±0.05 | 0.85 ±0.05 | 0.38±0.03 | 0.55 ±0.08 | 1.11 ±0.08 | 0.50 ±0.04 | 0.75 ±0.06 | 1.10 ±0.10 | 0.68 ±0.09 |
| NIL2 | 0.15 ±0.03 | 0.74 ±0.08 | 0.20 ±0.02 | 0.26 ±0.06 | 0.92 ±0.17 | 0.28 ±0.01 | 0.59 ±0.03 | 1.04 ±0.10 | 0.56 ±0.12 | 0.72 ±0.08 | 1.07 ±0.08 | 0.68 ±0.10 |
| NIL3 | 0.25 ±0.02 | 0.87 ±0.11 | 0.28 ±0.01 | 0.27 ±0.08 | 0.87 ±0.01 | 0.31 ±0.03 | 0.47 ±0.08 | 1.10 ±0.07 | 0.43 ±0.03 | 0.80 ±0.09 | 1.15 ±0.11 | 0.70 ±0.14 |
| NIL4 | 0.17 ±0.02 | 0.87 ±0.08 | 0.20 ±0.03 | 0.23 ±0.07 | 0.84 ±0.03 | 0.27 ±0.01 | 0.51 ±0.11 | 1.02 ±0.13 | 0.50 ±0.13 | 0.64 ±0.04 | 1.03 ±0.09 | 0.62 ±0.08 |
| NIL5 | 0.17 ±0.02 | 0.90 ±0.01 | 0.18 ±0.02 | 0.28 ±0.03 | 0.94 ±0.05 | 0.30 ±0.02 | 0.63 ±0.09 | 0.89 ±0.11 | 0.70 ±0.05 | 0.83 ±0.09 | 0.96 ±0.03 | 0.87 ±0.04 |
| NIL6 | 0.16 ±0.01 | 0.78 ±0.03 | 0.20 ±0.02 | 0.30 ±0.02 | 0.86 ±0.03 | 0.35 ±0.02 | 0.71 ±0.07 | 1.06 ±0.06 | 0.67 ±0.03 | 0.80 ±0.11 | 0.98 ±0.06 | 0.82 ±0.08 |
| NIL7 | 0.14 ±0.02 | 0.91 ±0.06 | 0.15 ±0.02 | 0.28 ±0.05 | 0.98 ±0.01 | 0.29 ±0.01 | 0.44 ±0.09 | 1.04 ±0.15 | 0.43 ±0.12 | 0.77 ±0.06 | 1.32 ±0.10 | 0.59 ±0.05 |
| NIL8 | 0.17 ±0.02 | 0.87 ±0.08 | 0.20 ±0.01 | 0.29 ±0.01 | 0.85 ±0.04 | 0.34 ±0.02 | 0.67 ±0.08 | 1.13 ±0.04 | 0.60 ±0.18 | 0.74 ±0.11 | 1.00 ±0.09 | 0.74 ±0.10 |
| NIL9 | 0.18 ±0.01 | 0.90 ±0.04 | 0.20 ±0.02 | 0.33 ±0.05 | 0.98 ±0.04 | 0.34 ±0.02 | 0.48 ±0.11 | 1.23 ±0.08 | 0.39 ±0.11 | 0.69 ±0.09 | 1.03 ±0.04 | 0.67 ±0.06 |
| NIL10 | 0.21 ±0.03 | 0.79 ±0.09 | 0.26 ±0.01 | 0.28 ±0.02 | 0.84 ±0.02 | 0.33 ±0.02 | 0.71 ±0.07 | 0.92 ±0.06 | 0.77 ±0.18 | 0.96 ±0.05 | 1.12 ±0.04 | 0.86 ±0.05 |
| NIL11 | 0.16 ±0.02 | 0.87 ±0.06 | 0.19 ±0.01 | 0.33 ±0.05 | 0.88 ±0.01 | 0.38 ±0.01 | 0.73 ±0.10 | 0.95 ±0.04 | 0.77 ±0.14 | 0.98 ±0.07 | 1.15 ±0.08 | 0.85 ±0.11 |
| NIL12 | 0.22 ±0.03 | 0.83 ±0.08 | 0.26 ±0.04 | 0.37 ±0.04 | 0.89 ±0.04 | 0.42 ±0.02 | 0.76 ±0.09 | 1.02 ±0.09 | 0.75 ±0.01 | 0.84 ±0.04 | 1.11 ±0.11 | 0.76 ±0.17 |
| NIL13 | 0.20 ±0.04 | 0.91 ±0.06 | 0.22 ±0.05 | 0.22 ±0.01 | 0.83 ±0.22 | 0.27 ±0.13 | 0.42 ±0.12 | 1.14 ±0.10 | 0.37 ±0.05 | 0.62 ±0.06 | 1.06 ±0.10 | 0.58 ±0.13 |
| NIL14 | 0.19 ±0.02 | 0.93 ±0.06 | 0.20 ±0.03 | 0.25 ±0.02 | 0.95 ±0.08 | 0.26 ±0.08 | 0.59 ±0.11 | 1.19 ±0.05 | 0.50 ±0.08 | 0.66 ±0.03 | 0.90 ±0.04 | 0.73 ±0.04 |
| NIL15 | 0.18 ±0.01 | 0.93 ±0.03 | 0.20 ±0.04 | 0.26 ±0.06 | 0.98 ±0.10 | 0.27 ±0.06 | 0.54 ±0.08 | 1.22 ±0.08 | 0.44 ±0.06 | 0.78 ±0.08 | 1.08 ±0.01 | 0.72 ±0.05 |
| NIL16 | 0.19 ±0.04 | 0.97 ±0.04 | 0.19 ±0.03 | 0.24 ±0.05 | 0.68 ±0.09 | 0.35 ±0.05 | 0.46 ±0.12 | 1.16 ±0.14 | 0.40 ±0.09 | 0.67 ±0.06 | 1.09 ±0.01 | 0.62 ±0.06 |
| NIL17 | 0.24 ±0.03 | 0.90 ±0.03 | 0.27 ±0.03 | 0.26 ±0.03 | 0.79 ±0.06 | 0.33 ±0.02 | 0.77 ±0.09 | 0.95 ±0.11 | 0.81 ±0.10 | 0.89 ±0.06 | 0.99 ±0.06 | 0.90 ±0.12 |
| NIL18 | 0.21 ±0.03 | 0.76 ±0.02 | 0.28 ±0.01 | 0.31 ±0.02 | 0.84 ±0.05 | 0.37 ±0.03 | 0.44 ±0.07 | 1.25 ±0.13 | 0.35 ±0.12 | 0.71 ±0.03 | 1.14 ±0.10 | 0.63 ±0.12 |
| NIL19 | 0.19 ±0.02 | 0.80 ±0.01 | 0.25 ±0.02 | 0.34 ±0.08 | 0.92 ±0.07 | 0.37 ±0.04 | 0.71 ±0.04 | 0.96 ±0.11 | 0.74 ±0.18 | 0.84 ±0.03 | 1.03 ±0.09 | 0.81 ±0.13 |
| NIL20 | 0.18 ±0.03 | 0.81 ±0.02 | 0.24 ±0.03 | 0.23 ±0.02 | 0.86 ±0.12 | 0.27 ±0.07 | 0.75 ±0.06 | 0.99 ±0.09 | 0.76 ±0.01 | 0.81 ±0.05 | 1.06 ±0.03 | 0.77 ±0.05 |
| PB 1509 | 0.17 ±0.02 | 0.81 ±0.04 | 0.23 ±0.02 | 0.22 ±0.07 | 0.85 ±0.09 | 0.26 ±0.05 | 2.19 ±0.03 | 0.47 ±0.03 | 4.62 ±0.15 | 3.33 ±0.16 | 0.45 ±0.05 | 7.37 ±0.18 |
| FL478 | 0.14 ±0.03 | 0.85 ±0.03 | 0.18 ±0.04 | 0.26 ±0.03 | 0.93 ±0.13 | 0.28 ±0.06 | 0.50 ±0.04 | 1.27 ±0.18 | 0.39 ±0.05 | 0.73 ±0.04 | 1.13 ±0.08 | 0.65 ±0.08 |
| **CD (0.05)** | **0.04** | **0.07** | **0.04** | **0.06** | **0.17** | **0.11** | **0.06** | **0.07** | **0.07** | **0.08** | **0.15** | **0.16** |

**Supplementary Table S5**: Na^+^ and K^+^ concentration PB 1509-NILs carrying *Saltol* QTL along with RP (PB 1509) and DP (FL478) under un-stressed and salt-stressed condition. Na^+^, sodium ion concentration in mmol/g of dry wt; K^+^, potassium ion concentration in mmol/g of dry wt; Na^+^/K^+^, sodium–potassium ratio; CD, critical difference.

**Supplementary Table S6**: Correlation coefficients among physiological traits MSI, RWC, Proline, ion content and salt tolerance under salt-stressed conditions. STS, salt tolerance score; MSI, membrane stability Index; RWC, leaf relative water content; Pro, proline content; Na^+^_St, sodium ion concentration in shoot in mmol/g; K^+^_St, potassium concentration in shoot in mmol/g; Na^+^/K^+^_St, sodium–potassium ratio in shoot; Na^+^_Rt, sodium ion concentration in root in mmol/g; K^+^_Rt, potassium concentration in root in mmol/g; Na^+^/K^+^_Rt, sodium–potassium ratio in root; *****Correlation coefficients are significant at *p* < 0.05 level; **Correlation coefficients are significant at *p* < 0.01 level.

| *Parameters* | *STS* | *MSI%* | *RWC%* | *Pro* | *Na^+^_St* | *K^+^_St* | *Na^+^/K^+^_St* | *Na^+^_Rt* | *K^+^_Rt* | *Na^+^/K^+^_Rt* |
| --- | --- | --- | --- | --- | --- | --- | --- | --- | --- | --- |
| *STS* | 1 |  |  |  |  |  |  |  |  |  |
| *MSI%* | -0.599** | 1.000 |  |  |  |  |  |  |  |  |
| *RWC%* | -0.917** | 0.719** | 1.000 |  |  |  |  |  |  |  |
| *Proline* | -0.535** | 0.686** | 0.713** | 1.000 |  |  |  |  |  |  |
| *Na^+^_St* | 0.879** | -0.638** | -0.814** | -0.475* | 1.000 |  |  |  |  |  |
| *K^+^_St* | -0.706** | 0.221 | 0.523 | 0.101 | -0.725** | 1.000 |  |  |  |  |
| *Na^+^/K^+^_St* | 0.956** | -0.599** | -0.862** | -0.469* | 0.971** | -0.803** | 1.000 |  |  |  |
| *Na^+^_Rt* | 0.928** | -0.631** | -0.910** | -0.624** | 0.786** | -0.561** | 0.858** | 1.000 |  |  |
| *K^+^_Rt* | -0.637** | 0.502* | 0.490* | 0.194 | -0.607** | 0.354 | -0.618** | -0.515* | 1.000 |  |
| *Na^+^/K^+^_Rt* | 0.981** | -0.640** | -0.915** | -0.569** | 0.850** | -0.632** | 0.922** | 0.971** | -0.672** | 1.000 |
